# PNUTS:PP1 recruitment to Tox4 regulates chromosomal dispersal in *Drosophila* germline development

**DOI:** 10.1016/j.celrep.2025.115693

**Published:** 2025-05-09

**Authors:** Louise Duncalf, Xinru Wang, Abdulrahman A. Aljabri, Amy E. Campbell, Rawan Q. Alharbi, Ian Donaldson, Andrew Hayes, Wolfgang Peti, Rebecca Page, Daimark Bennett

**Affiliations:** 1 Faculty of Health and Life Sciences, University of Liverpool, Biosciences Building, Crown Street, L69 7ZB Liverpool, UK; 2 Department of Molecular Biology, Cell Biology and Biochemistry, Brown University, Providence, RI, USA; 3 Faculty of Biology, Medicine and Health, University of Manchester, Michael Smith Building, Oxford Road, M13 9PT Manchester, UK; 4 Department of Pharmacology and Toxicology, College of Pharmacy, Taibah University, Madinah, Kingdom of Saudi Arabia; 5 Department of Molecular Biology and Biophysics, University of Connecticut Health Center, Farmington, CT, USA; 6 Department of Cell Biology, University of Connecticut Health Center, Farmington, CT, USA; 7 Present address: The Hewitt Fertility Centre, Liverpool Women’s Hospital, Crown Street, Liverpool, L8 7SS Merseyside, UK; 8 Present address: Institute for Protein Design, University of Washington, Seattle, WA, USA; 9 Present address: Kinomica, Mereside, Alderley Park, Alderley Edge, SK10 4TG Macclesfield, UK; 10 Lead contact

## Abstract

Ser/Thr protein phosphatase 1 (PP1) forms a large nuclear holoenzyme (with PNUTS, WDR82, and Tox4) whose emerging role is to regulate transcription. However, the role of Tox4, and its interplay with the other phosphatase subunits in this complex, is poorly understood. Here, we combine biochemical, structural, cellular, and *in vivo* experiments to show that, while *tox4* is dispensable for viability, it is essential for fertility, having both PNUTS-dependent and -independent roles in *Drosophila* germline development. We also show that Tox4 requires zinc for PNUTS TFIIS N-terminal domain (TND) binding, and that it binds the TND on a surface distinct from that used by established TND-interacting transcriptional regulators. We also show that selective disruption of the PNUTS-Tox4 and the PNUTS-PP1 interaction is critical for normal gene expression and chromosomal dispersal during oogenesis. Together, these data demonstrate how interactions within the PNUTS-Tox4-PP1 phosphatase combine to tune transcriptional outputs driving developmental transitions.

## In brief

Duncalf et al. investigate the evolutionarily conserved interaction between the PNUTS N-terminal domain and the TOX4 C-terminal domain, resolving the PNUTS:TOX4 complex crystal structure. Leveraging these insights, the authors define the functional significance of Tox4-PNUTS-PP1 interactions in *Drosophila* germline development, highlighting their cooperative role in developmentally controlled chromosome reorganization.

## Graphical Abstract

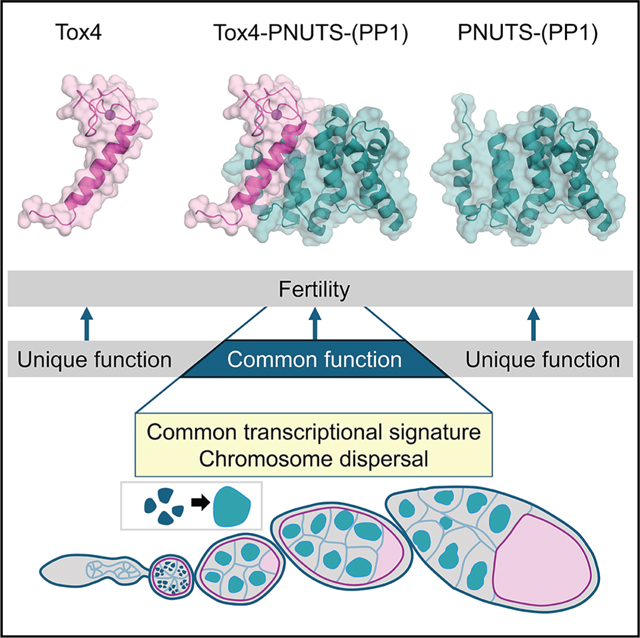

## INTRODUCTION

Multivalent binding proteins play vital roles in recruiting regulatory enzymes and effectors at defined cellular sites to ensure specific biological outcomes. During eukaryotic gene expression, multiprotein phosphatase-containing complexes are essential to control RNA polymerase II (RNAPII) at sites of transcription, particularly to ensure transcription cycle phase transitions, such as regulating the release from polymerase pausing during early elongation and ensuring productive mRNA synthesis.^1,2^ This is because many transcription factors are functionally regulated by (de)phosphorylation. In addition, the reversible phosphorylation of multiple residues in the RNAPII C-terminal domain (RNAPII-CTD) controls its association with distinct factors that serve to coordinate elongation with co-transcriptional RNA processing.^3,4^

Protein phosphatase 1 (PP1) is the most widely expressed and abundant serine/threonine phosphatase, with roles during multiple steps of mRNA synthesis, as well as many other unrelated processes such as protein synthesis, muscle contraction, and carbohydrate metabolism.^5–8^ It achieves high specificity by interacting with more than 200 distinct regulatory proteins.^7,9–11^ The PP1 nuclear targeting subunit (PNUTS, also known as PPP1R10/p99/FB19/CAT53), which is one of the most abundant regulatory proteins of PP1 in the nucleus,^12,13^ has emerged as an essential regulator of gene expression. PNUTS regulates multiple steps of mRNA synthesis to control RNAPII pause release,^14,15^ block promiscuous initiation,^16,17^ facilitate RNA splicing,^18^ and promote transcription termination.^19,20^ In addition to targeting RNAPII-CTD Ser5 for dephosphorylation by PP1,^20–22^ PNUTS also regulates the functionally critical phosphorylation state of transcription factors, SPT5,^19^ p53^23^, and MYC.^24^ PNUTS also regulates retinoblastoma (Rb) protein in response to cellular stress,^25,26^ and associates with chromatin to promote chromosome decondensation,^27^ mitotic exit,^28^ and DNA damage responses.^29–32^ How PNUTS, via PP1 scaffolding, mediates these distinct functions remains a largely open question.

**Figure 1.**
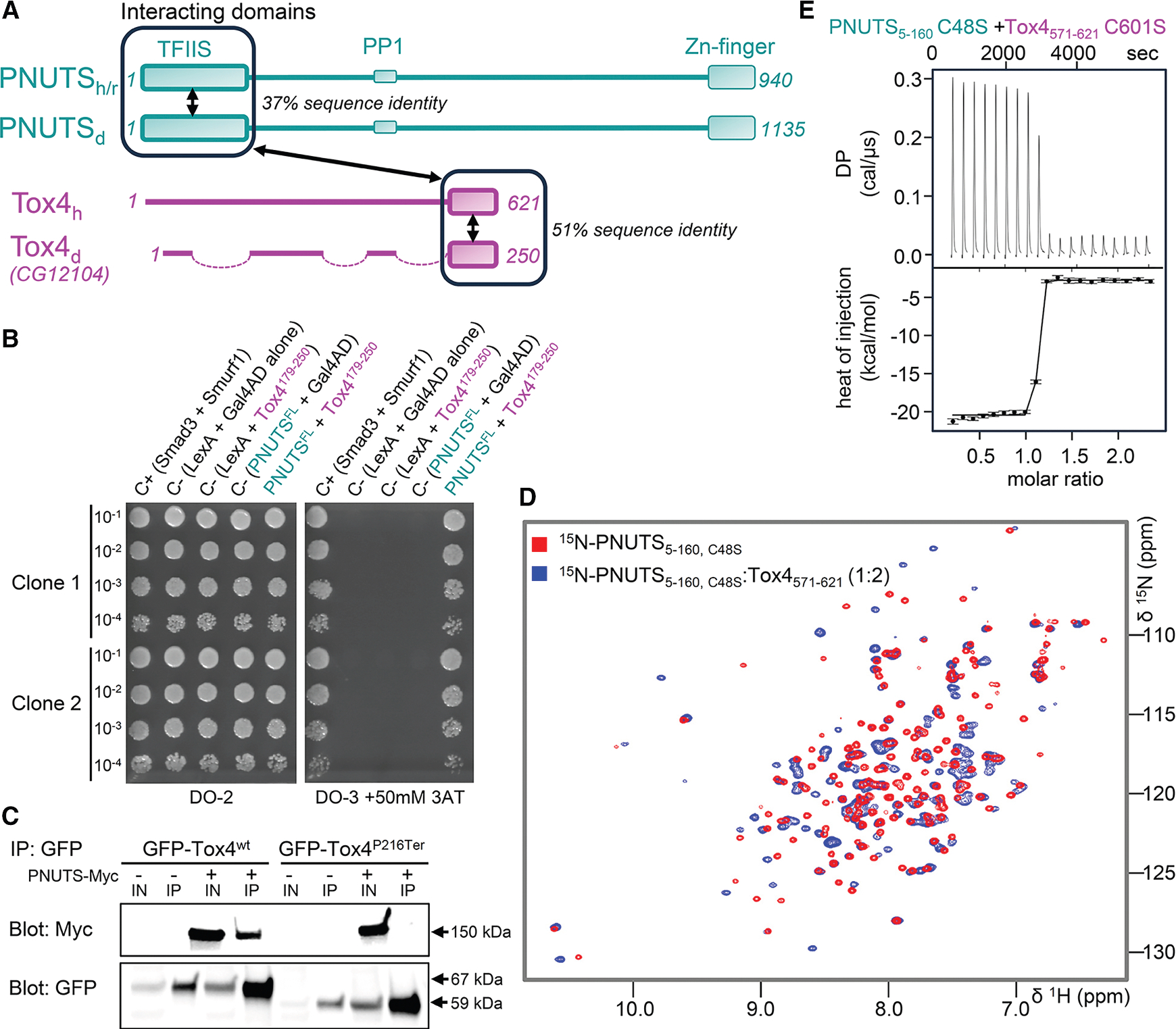
Evolutionarily conserved interaction between PNUTS N-terminal domain and Tox4 C-terminal domain (A) Domain organization of mammalian (human/rat) and fly (*Drosophila*) PNUTS (aqua) and Tox4 (pink), with key domains labeled. Sequence identity between mammalian and fly for the PNUTS N-terminal TFIIS domain and the Tox4 C-terminal domain are indicated. Domains that interact between PNUTS and Tox4 are shown by a double arrowed line. (B) *Drosophila* PNUTS interacts with Tox4 in the yeast two-hybrid system. PNUTS full-length bait (PNUTS^1–1135^-LexA DNA binding domain) and Tox4 prey (Tox4^179–250^-Gal4 activation domain) plasmids were tested in duplicate (clones 1 and 2) at several dilutions, as indicated. Auxotrophic growth on media without tryptophan and leucine (DO-2) or without tryptophan, leucine, and histidine with 50 mM 3-aminotriazol (DO-3+ 3-AT) is shown. C+, positive control with interacting bait and prey plasmids for Smad3 and Smurf1, respectively; C–, negative controls, as indicated. (C) Binding of Myc-tagged PNUTS to GFP-tagged Tox4 requires the Tox4 C terminus (residues 216–250) in pull-downs from *Drosophila* S2R+ cells. Immunoblots show total protein extract (IN) and immunoprecipitated protein extract (IP) from S2R+ cells co-transfected with GFP-tagged wild-type (Tox4^wt^) or truncated (Tox4^P216Term^) Tox4, with or without myc-tagged PNUTS. (D) 2D [^1^H,^15^N]HSQC spectrum of ^15^N-labeled PNUTS_5–160_ C48S alone (red) and in complex with Tox4_571–621_ (blue). (E) Binding isotherm of PNUTS_5–160_ C48S with Tox4_571–621_ C601S.

Central to PNUTS function within the PNUTS:PP1 phosphatase holoenzyme complex is its role as protein interaction hub. PNUTS, a largely intrinsically disordered protein, contains multiple functional units/domains, in addition to its central PP1 binding domain (residues 394–433). These include a folded transcriptional elongation factor S-II N-terminal homology domain (TFIIS TND; residues 1–160), an intrinsically disordered region (IDR) WDR82-interaction domain, C-terminal RGG RNA-binding repeats (residues 850–900), and a zinc finger domain (residues 900–940) (Figure 1A). Its TND has emerged as a key transcription assembly module that interacts directly and reversibly with a set of cognate disordered ligands known as TND-interacting motifs (TIMs) present in the transcription factor MYC,^33^ as well as transcription elongation regulators including IWS1, SPT6, and PAF1.^34^ Via these domains, PNUTS scaffolds a constitutive complex containing Tox4, Wdr82, and PP1, hereafter referred to as the PTW:PP1 complex.^35^ PTW:PP1 complex formation allows for the recruitment of RNAPII-CTD Ser5^36^ and stimulates the RNAPII-CTD dephosphorylation by PP1 *in vivo.*^20^ Wdr82 is a structured WD40 domain protein that binds PNUTS just C-terminal to its PP1 binding domain.^20,35^ Tox4 is predicted to be a largely unstructured member of the DNA-binding high-mobility group box protein family (Figure 1A), which has been reported to restrict RNAPII pause release and early elongation, promote late elongation, and facilitate transcriptional reinitiation.^37,38^ However, in the absence of molecular information, how PNUTS assembles Tox4 with other PTW:PP1 components into functional transcription complexes remains under-investigated.

Our previous data showed that the PNUTS:PP1 holoenzyme is necessary for developmental gene expression,^21,39^ but the identity of its interacting partners and their contribution to PNUTS function *in vivo* was not known. Here, we identify *Drosophila* Tox4 as a PNUTS binding protein, show that it binds zinc, and demonstrate that zinc binding is critical for the formation of the PNUTS:Tox4 complex. Our crystal structure of the Tox4: PNUTS complex shows that interaction between these proteins is extensive and, as we show using binding affinity measurements, constitutive, consistent with the previous observations that the PTW:PP1 complex is stable throughout the cell cycle. We also show that, while *Drosophila tox4* is dispensable for viability, it is essential for fertility, having both *PNUTS*-dependent and -independent roles in *Drosophila* germline development. Finally, using structure-based mutagenesis, we show that integration of both functions by PNUTS is necessary for normal gene expression and germline development. Collectively, these findings provide insight into the function of Tox4, both independently and in coordination with other PTW:PP1 components, in shaping transcriptional programs during phases of cellular and tissue maturation.

## RESULTS

### Tox4:PNUTS binding is evolutionarily conserved in *Drosophila*

We identified *Drosophila CG12104*, which encodes the only *Drosophila* Tox4 homolog,^40^ in a yeast two-hybrid (Y2H) screen for *Drosophila* PNUTS-interacting proteins. Domain analysis of 61 recovered *tox4* Y2H cDNA clones showed that the PNUTS-interacting region mapped to the Tox4 C terminus (residues 206–246) (Figure 1B), consistent with previous deletion co-immunoprecipitation studies showing that the C terminus of human Tox4 binds to the N terminus of PNUTS.^35^ These data confirm the evolutionarily conserved nature of PNUTS:Tox4 interactions. To further validate the importance of the Tox4 C terminus for binding, *Drosophila* S2R+ cells were transiently transfected with Myc-tagged *Drosophila* PNUTS and GFP-tagged *Drosophila* Tox4^wt^ or GFP-Tox4^P216Ter^, which lacks the last 34 residues of Tox4. Blotting with an anti-GFP antibody confirmed expression of GFP-Tox4 in cell lysates. GFP-Tox^wt^, but not GFP-Tox4^P216Ter^, co-precipitated with Myc-tagged PNUTS (Figure 1C). Together, these data confirm that the C terminus of *Drosophila* Tox4 is required for PNUTS binding in *Drosophila* S2R+ cells.

### The C-terminal domain of Tox4 is a zinc binding domain that binds constitutively to PNUTS

We next tested the interaction of the PNUTS TND with the C-terminal domain of Tox4 *in vitro*. PNUTS TND domain constructs (rat, aa 1–160, 5–160; Figure 1A) are readily expressed in *E. coli*. The high-quality 2D [^1^H,^15^N]HSQC spectrum of PNUTS_TND_ (for NMR spectroscopy and X-ray crystallography, PNUTS construct 5–160 [C48S] was used throughout the study; Figure 1D) showed that the PNUTS_TND_ is well folded. In contrast, while Tox4_CTD_ (human Tox4_571–621_) purifies readily, its 2D [^1^H,^15^N]HSQC spectrum was of low quality with many peaks showing significant broadening (Figure S1). After exhausting canonical strategies to improve the NMR spectrum, we used the fold and function assignment system (FFAS)^41^ to identify proteins that are predicted to exhibit structural homology to Tox4_CTD_. FFAS predicted a weak homology to the MYND zinc binding domain (15% sequence identity) and the zinc finger HIT domain in the DEAD box polypeptide 59 (11% sequence identity), suggesting that Tox4_CTD_ might also bind zinc. To test if Tox4_CTD_ binds zinc, we measured the 2D [^1^H,^15^N]HSQC spectrum of the Tox4_CTD_ in the presence of zinc, which substantially improved the spectrum (Figure S1), demonstrating that the fold of Tox4_CTD_ is stabilized by zinc binding. We then titrated a 1:2 M ratio of zinc-loaded Tox4 to ^15^N-labeled PNUTS_TND_. The 2D [^1^H,^15^N]HSQC spectrum of PNUTS_TND_ showed dramatic chemical shift perturbations (CSPs), confirming a direct interaction between the two proteins (Figure 1D). Finally, subsequent isothermal titration calorimetry (ITC) experiments showed that Tox4_CTD_ and PNUTS_TND_ bind tightly, with a K_D_ = 0.3 ± 0.1 nM (Figures 1E and S2; Table S1).

### Crystal structure of the PNUTS_TND_:Tox4_CTD_ complex

**Figure 2.**
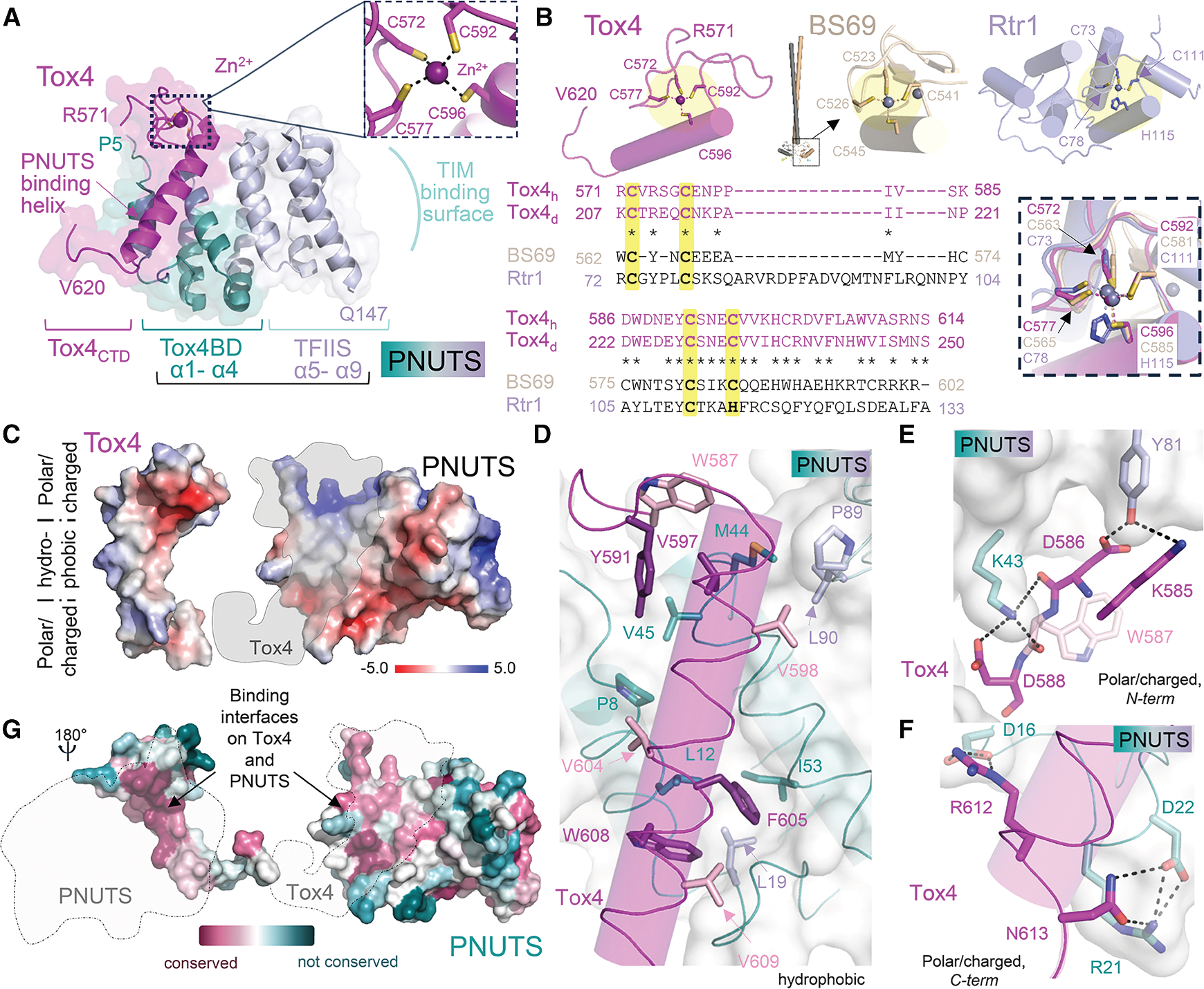
Crystal structure of the PNUTS:Tox4 complex (A) Structure of the Tox4 (magenta) and PNUTS NTD (Tox4 binding domain [Tox4BD], helices ɑ1-ɑ4, dark teal; Tox4 TFIIS domain, helices ɑ5-ɑ9, light teal) complex. The Tox4 PNUTS binding helix and TND-interacting motif (TIM) surface on PNUTS are labeled. Dotted box is Tox4 zinc binding pocket, shown also in the inset with zinc binding residues labeled. (B) A DALI structure similarity search identified RTR1 (lavender; PDB: 4FC8) and BS69 (gray; PDB: 5C2Y) as having weak similarity with the Tox4 C-terminal domain. Tox4, BS69, and Rtr1 structures, respectively, are shown at the top. BS69 is a dimer with the zinc binding domain at the end of a long coiled coil. Lower panel, structure-based sequence alignment of Tox4 (both the human and *Drosophila* sequence shown with identical residues indicated by an “*”), BS69, and Rtr1, with DALI *Z* scores reported, together with overlay of the zinc binding pockets with zinc binding residues labeled. (C) Interaction surfaces of Tox4 and PNUTS showing the electrostatic potential surface, with the hydrophobic (middle) and polar/charged (upper/lower) regions labeled. (D) Hydrophobic interactions between Tox4 (magenta/pink) and PNUTS (dark teal/light teal). Residues that become nearly completely buried upon complex formation are colored in dark shades. (E) N-terminal polar/charged interaction surface, with hydrogen bonds/salt bridges between Tox4 and PNUTS shown as dashed lines. (F) C-terminal polar/charged interaction surface, with inter- and intramolecular hydrogen bonds/salt bridges shown as dashed lines. (G) Consurf analysis of Tox4 (left) and PNUTS (right), with the regions at the PNUTS:Tox4 interface indicated by gray dotted lines.

To define how Tox4_CTD_ binds PNUTS_TND_, we determined the crystal structure of the *PNUTS*_*TND*_*:Tox4*_*CTD*_ complex (Figure 2A; Table S2; 2.1 Å resolution; PNUTS_TND_ [5–160, C48S] and Tox4_CTD_ [571–621, C601S], hereafter referred to as PNUTS: Tox4 complex). PNUTS adopts a nine α-helical bundle structure consistent with the PNUTS_*TND*_ solution structures^33,34^; its structure is most similar to the IWS1/Spn1 protein (DALI *Z* scores = 13.4–13.8).^42^ PNUTS helices 5–9 form the TFIIS N-terminal domain (TND), which is a conserved scaffold enriched in transcription factors that mediates binding with TND-interacting motifs (TIMs) and is the domain that exhibits the greatest overlap with IWS1 (Figure 2A). This domain has previously been shown to bind directly to the MYC TIM (_34_VQPYF_38_) in addition to TIMs from other transcription factors (including SPT6 and PAF1).^34^ Helices 1–4, which pack tightly against the TFIIS domain to form a single compact fold, adopt a distinct α-helical bundle that is not present in other known TFIIS TND-containing proteins. It is this domain, referred to as the Tox4 binding domain (Tox4BD), that binds the Tox4_CTD_ (Figure 2A).

The Tox4_CTD_ adopts a novel conformation, where aa 571–596 form a zinc binding loop in which Tox4 residues Cys572, Cys577, Cys592, and Cys596 coordinate a zinc ion, while residues 597–612 form a long α helix (the Tox4 PNUTS binding helix) (Figures 2A, 2B, and S3). A structure similarity search using DALI identified only weakly similar proteins (*Z* scores = 2.0–2.7), including RTR1, a putative atypical phosphatase that regulates the phosphorylation status of the RNAPII-CTD (*Z* score = 2.7), and the BS69/ZMYND11 protein that contains an MYND zinc binding domain (*Z* score = 2.2) (Figure 2B). While the zinc binding residues overlap between these proteins, the conformations of the intervening loops and the residues beyond the zinc binding domain differ, explaining the low similarity scores (Figure 2B).

The binding interface between Tox4 and PNUTS is extensive, with the Tox4 PNUTS binding helix interacting with PNUTS α helices α1 and α3 via hydrophobic and ionic interactions (Figure 2C). The hydrophobic interactions are toward the center of the Tox4-PNUTS interface (Figure 2D; *Tox4*: W587_Tox4_, Tyr591_Tox4_, Val597_Tox4_, Val598_Tox4_, Val604_Tox4_, Phe605_Tox4_, Trp608_Tox4_, V609_Tox4_; *PNUTS*: Pro8_PNUTS_, Leu12_PNUTS_, Leu19_PNUTS_, Met44_PNUTS_, Val45_PNUTS_, Ile53_PNUTS_, Pro89_PNUTS_, Leu90_PNUTS_). These interactions are bounded at both ends by polar/electrostatic contacts (Figures 2E and 2F; *N-term*: Lys585_Tox4_, Asp586_Tox4_, Asp588_Tox4_ with Lys43_PNUTS_, Tyr81_PNUTS_; *C-term*: Arg612_Tox4_, Asn613_Tox4_ with Asp16_PNUTS_, Arg21_PNUTS_, Asp22_PNUTS_). Finally, the aromatic ring of Trp587_Tox4_ forming an aromatic-sulfur interaction with Met44_PNUTS_ (Figure 2D) and Lys585_Tox4_ forms a cation-π interaction with Trp81_PNUTS_ (Figure 2E).

These interacting residues are conserved in *Drosophila* PNUTS_TND_ and Tox4_CTD_ sequences (Figures 2B and S4). To test if the residues that mediate binding are more extensively conserved, we performed a ConSurf analysis.^43,44^ The analysis showed that PNUTS residues in α helix 1, the α helix 1/2 linker region and α helix 3, which are key for forming the novel conserved interaction surface in the PNUTS_TND_ that so far is exclusively used by Tox4, are highly conserved (Figure 2G). Likewise, the TOX4 residues that mediate PNUTS binding are equally conserved (Figure 2G). Finally, consistent with its nanomolar binding affinity, the complex buries 2,100 Å^2^ of solvent-accessible surface area. This tight, extensive interaction, coupled with that observed between PNUTS and PP1,^25^ explain why PNUTS, Tox4, and PP1 form a constitutive complex in cells.

### Accumulation of Tox4 in the nucleus depends on PNUTS binding

**Figure 3.**
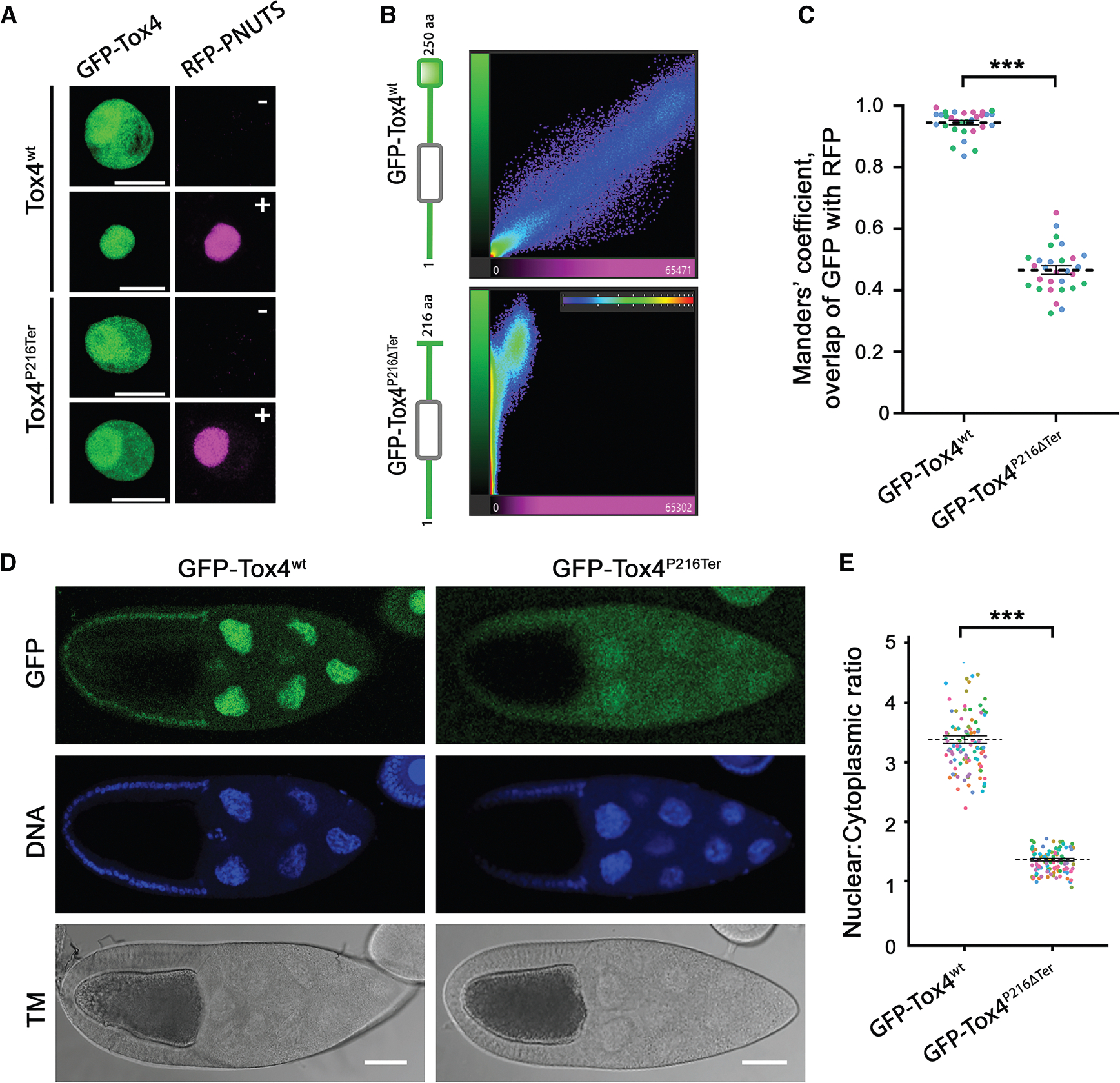
PNUTS facilitates accumulation of Tox4 in the nucleus *in vitro* and *in vivo* (A) Maximal projection of stack of confocal images showing subcellular localization of ectopically expressed GFP-tagged Tox4 and RFP-tagged PNUTS throughout live S2R+ cells. In the absence of RFP-PNUTS (−), GFP-Tox4^wt^, and GFP-Tox4^P216Term^ are found in both the nucleus and cytoplasm. In the presence of RFP-PNUTS (+), GFP-Tox4^wt^ accumulates in the nucleus, whereas the distribution of GFP-Tox4^P216Term^ is largely unaffected. Scale bars, 10 μm. (B) Intensity histograms showing distribution of pairs of GFP and RFP voxel intensities of cells in (A) co-transfected with GFP-Tox4 (green) and RFP-PNUTS (magenta), revealing strong colocalization of RFP-PNUTS and GFP-Tox4^wt^ but not GFP-Tox4^P216Term^. (C) Plot showing mean ± SEM of Manders’ coefficient of the overlap between GFP and RFP in cotransfected cells. Means are derived from *n* = 3 experiments, shown on Beeswarm plots of individual measurements of repeated counts (*n* = 10 cells/biological replicate). (D) Distribution of GFP-tagged Tox4 under the control of the endogenous tox4 promoter in *Drosophila* stage 10 egg chambers. Fluorescent signal from GFP-Tox4^wt^ (green) is clearly evident in both (smaller) somatic and (larger, polyploid) nurse cell nuclei, costained for DNA with DAPI (blue). In contrast, the signal from GFP-Tox4^P216Term^ can be seen less strongly in the nucleus and is diffusely localized throughout the cytoplasm. (E) Quantification of nuclear/cytoplasmic ratio. Datapoints for individual nuclei, color coded by egg chamber, are shown together with mean ± SEM (*n* = 21 egg chambers, ~100 nuclei/genotype. Scale bars, 50 μm. Statistical significance was tested using pairwise t tests with Bonferroni *p* value correction. ***p < 0.001.

To further investigate Tox4:PNUTS complex formation in cells, we examined the subcellular distribution of Tox4 and PNUTS. *Drosophila* S2R+ cells were transiently co-transfected with GFP-tagged *Drosophila* Tox4^wt^ or Tox4^P216Ter^, alone or together with RFP- *Drosophila* PNUTS, and imaged using confocal microscopy. Single transfections revealed that GFP-Tox4^wt^ and -Tox4^P216Ter^ were distributed in both the nucleus and the cytoplasm, with a higher level of expression in the nucleus (Figure 3A). Upon co-transfection with RFP-PNUTS, GFP-Tox4^wt^ became enriched in the nucleus and strongly co-localized with RFP-PNUTS (Manders’ coefficient = 0.945 ± 0.009) whereas GFP-Tox4^P216Ter^ did not (Manders’ coefficient = 0.466 ± 0.010) (Figures 3B and 3C). We then examined the subcellular distribution of *Drosophila* Tox4 *in vivo* using GFP-tagged Tox4^wt^ or Tox4^P216Ter^ expressed from transgenic constructs under the control of the endogenous *tox4* promoter. A difference in localization of GFP-tagged Tox4^wt^ or Tox4^P216Ter^ was clearly visible (Figure 3D). In nurse cells in the ovary, GFP-Tox4^wt^ showed a 3.3-fold enrichment in nuclei relative to the cytoplasm (*p* < 0.001), whereas GFP-Tox4^P216Ter^ showed a more diffuse distribution (mean nuclear/cytoplasmic ratio 1.31, Figures 3D and 3E). Taken together, these data confirm that PNUTS is critical for nuclear accumulation of Tox4.

### Tox4 and PNUTS-Tox4 binding are dispensable for viability

**Figure 4.**
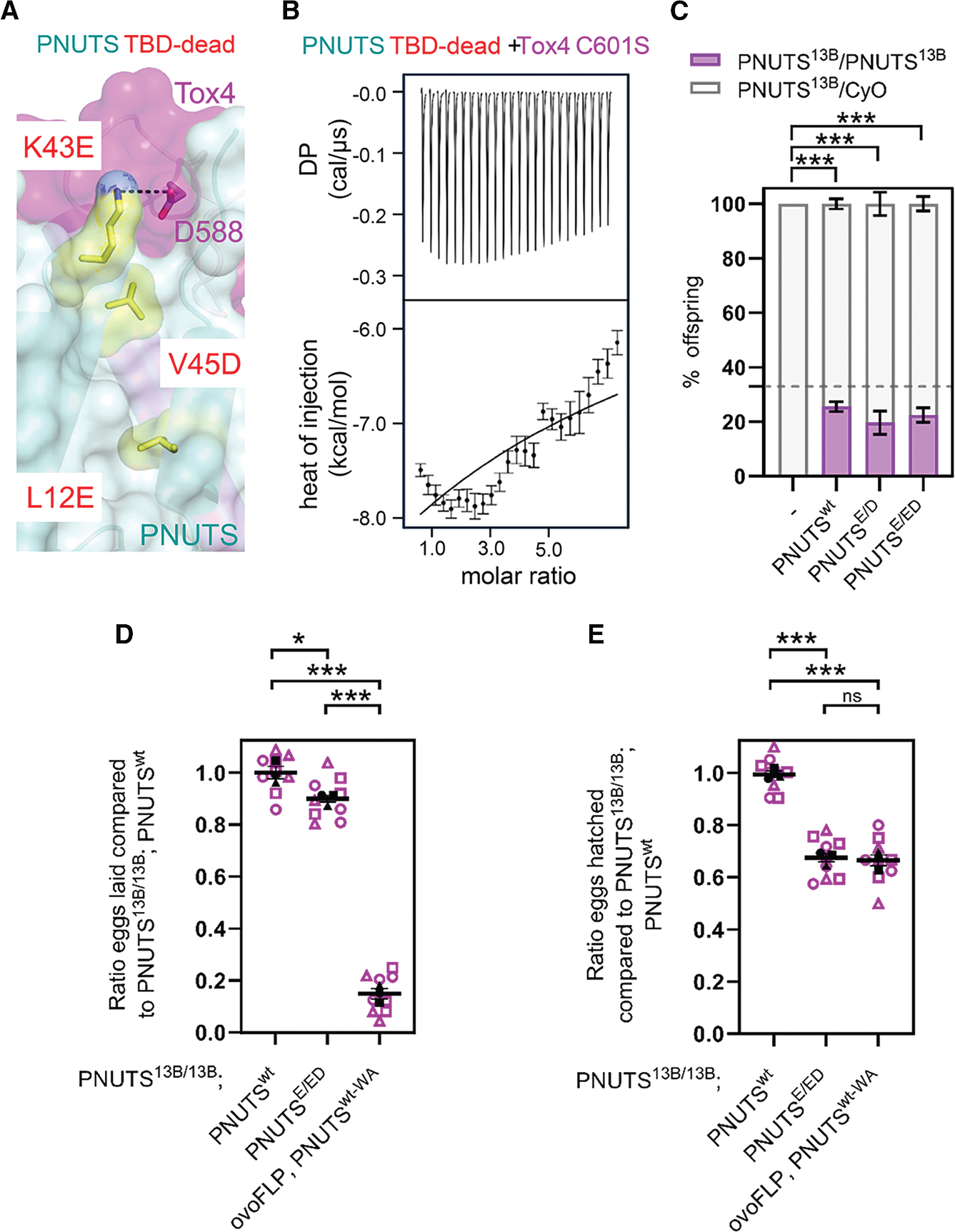
Tox4 binding to PNUTS is not essential for viability (A) PNUTS:Tox4 interaction interface highlighted the residue mutations to generate PNUTS_TBD-dead_—L12E, K43E, and V45D—shown in yellow. (B) Representative binding isotherm of PNUTS_TBD-dead_ with Tox4_571–621_ C601S. (C) Results of complementation tests with genomic *PNUTS* transgenes to determine their ability to rescue larval lethality of *PNUTS*^*13B*^ homozygous animals. A prediction of how often this genotype should be represented if there were no mutation (corresponding to a Mendelian ratio of 2:1 heterozygous/homozygous animals) is shown with a dotted line. Wild-type (PNUTS^wt^) and reduced-Tox4 binding mutants (PNUTS^ED^ and PNUTS^E/ED^) all rescued *PNUTS*^*13B*^ homozygotes to adulthood (mean ± SEM is shown, *n* = 5). (D) Quantitation of ratio of eggs laid by the indicated genotype of parental flies relative to control flies (*PNUTS*^*13B/13B*^*; PNUTS*^*wt*^). Egg laying in *PNUTS*^*13B/13B*^ flies was strongly reduced by inducible *PNUTS*^*wt-flp-W726A*^ and weakly reduced by *PNUTS*^*E/ED*^. (E) Quantitation of ratio of eggs from the indicated genotype of parental flies that hatched relative to control. Hatching was reduced to a similar extent by *PNUTS*^*wt-flp-W726A*^ and *PNUTS*^*E/ED*^. (D and E) Plots show overall mean ± SEM derived from means of *n* = 3 experiments, superimposed on Beeswarm plots of individual measurements of repeated counts for each of the indicated genotypes. ns, not significant; *p < 0.05, **p < 0.01, ***p < 0.001 by one-way ANOVA.

To establish the role(s) of the Tox4-PNUTS interaction, we tested if the Tox4:PNUTS complex can be disrupted by mutagenesis. Using the crystal structure, we engineered a PNUTS Tox4 binding domain dead variant (PNUTS_TBD-dead_) with the following mutations: K43E_PNUTS_ (charge reversal to disrupt the salt bridge with D586_Tox4_), L12E_PNUTS_, and V45D_PNUTS_ (hydrophobic residues that are completely buried at the PNUTS-Tox4 hydrophobic interface) (Figure 4A). PNUTS_TBD-dead_ expressed and purified readily with subsequent ITC experiments demonstrating that it no longer binds Tox4 (Figure 4B; Table S1).

To test the *in vivo* effect of disrupting the interaction between PNUTS and Tox4, we generated GFP-tagged *Drosophila* PNUTS rescue constructs, expressed under control of the endogenous PNUTS promoter, with the structurally derived and tested mutations in the Tox4 binding pocket (PNUTS^ED^-GFP [K43E/V45D] and PNUTS^E/ED^-GFP [K43E/L12E/V45D]) (Figure S5). Expression of wild-type PNUTS-GFP in flies largely rescued a recessive lethal *PNUTS* null mutant (*PNUTS*^*13B*^)^21^ (Figure 4C). One or two copies of either PNUTS^ED^ or PNUTS^E/ED^ also rescued the lethality of *PNUTS* loss of function (Figure 4C). PNUTS^E/ED^ was at least as well tolerated as PNUTS^ED^, and there was no significant difference in the extent of rescue between PNUTS^E/ED^ and *PNUTS*^wt^. Together, these data show that PNUTS-Tox4 binding is dispensable for viability. When we tested the fertility of surviving adult female flies, we observed a modest but significant reduction in both egg laying (Figure 4D) and egg hatching (Figure 4E), indicating a reduction in egg production and viability.

Since PNUTS scaffolds both Tox4 and PP1 in a stable complex,^35^ we wondered whether PNUTS-PP1 binding was also necessary for female fertility. To test this, we used a conditional transgenic PNUTS allele (*PNUTS*^*wt-flp-W726A*^) that undergoes an irreversible transition from wild-type PNUTS (PNUTS^wt^) to a PP1 binding-incompetent version (PNUTS^W726A^)^21^ after FRT/flp-induced allele exchange (Figure S6A). When we specifically induced exchange of PNUTS^wt^ to PNUTS^W726A^ in all ovarian germline cells (Figure S6B), we found a much stronger reduction in egg laying than in mutant *PNUTS*^*E/ED*^ animals, as well as a reduction in hatching (Figures 4D and 4E), suggesting that PNUTS:PP1 may have roles independent of Tox4 binding in germline development.

### Tox4 is required for male and female fertility in *Drosophila*

**Figure 5.**
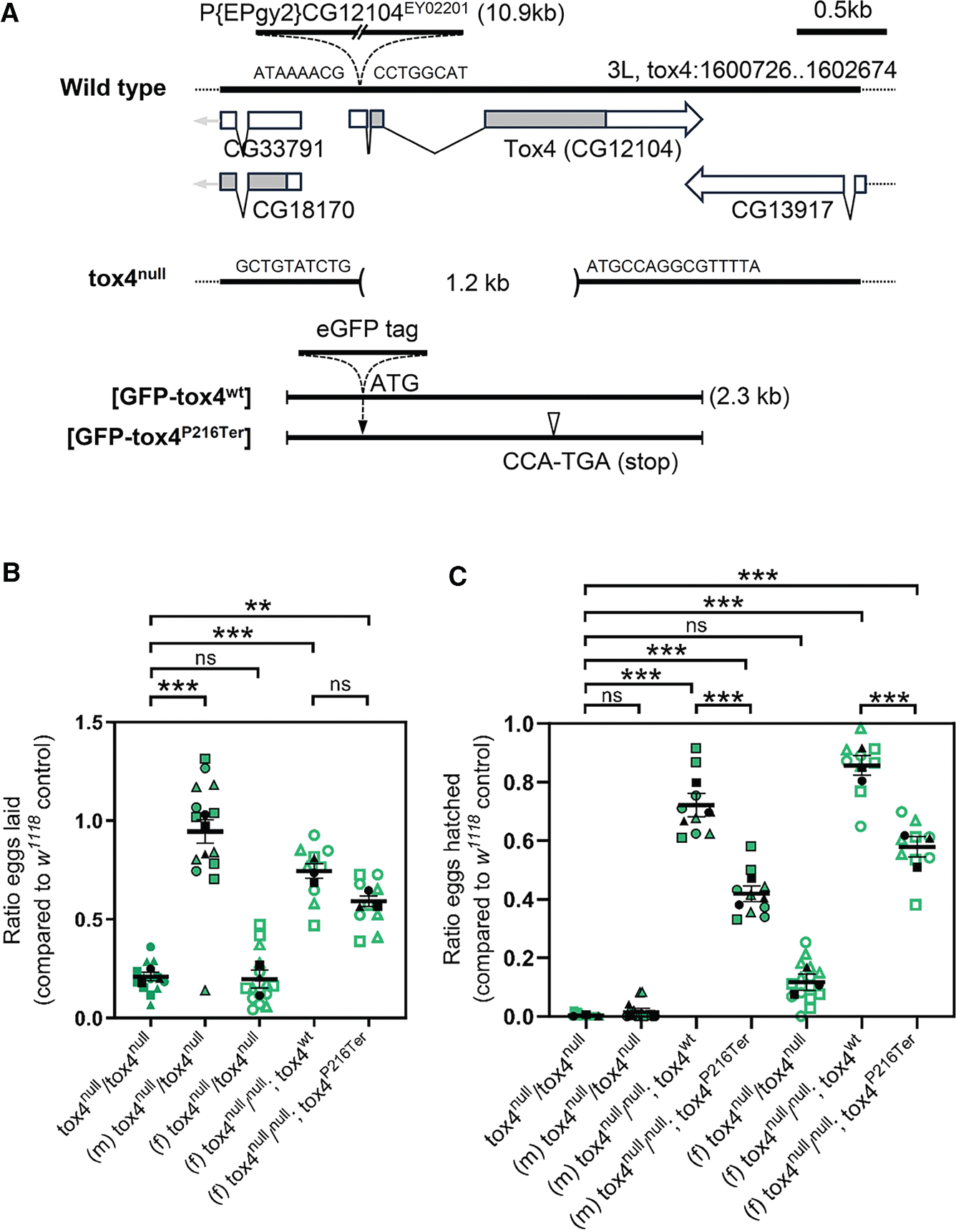
Tox4 is required for male and female fertility in *Drosophila* (A) Genomic region of *CG12104* (*Drosophila tox4*) showing exon-intron structure for *CG12104* and location of flanking genes. Gray shading represents coding regions, unfilled boxes represent untranslated regions with arrows indicating direction of transcription. *CG12104*^*EY02201*^ contains a *P* element insertion in the 5′ untranslated region of *tox4*. A 1.2 kb deletion of the *CG12104* coding sequence in *tox4*^*null*^ resulting from imprecise excision of *CG12104*^*EY02201*^ is indicated, together with genomic sequence of the breakpoints. Also shown is the extent of genomic transgenes carrying either wild-type or mutant *tox4* (*tox4*^*P216Term*^) transcription unit tagged at the N terminus with GFP. The base pair change in the transgenic GFP-tox4^P215Term^ allele is shown. (B) Quantitation of ratio of eggs laid by the indicated genotype of parental flies relative to controls (*w*^*1118*^), showing an 80% reduction in number in eggs from *tox4*^*null*^ mothers, and transgenic rescue of this effect with GFP-tox4. (m) male and (f) female parental flies were crossed to *w*^*1118*^ flies. Plots show overall mean ± SEM derived from means of *n* = 3 experiments superimposed on Beeswarm plots of individual measurements of repeated egg counts over at least 3 days/genotype. (C) Quantitation of ratio of eggs from the indicated genotype of parental flies that hatched relative to controls (*w*^*1118*^). Hatching was greatly reduced by loss of *tox4* function in male and female parental flies. This was rescued by transgenic *GFP-tox4*, with *GFP-tox4*^*wt*^ rescuing more strongly than *GFP-tox4*^*P215Term*^. Plots show overall mean ± SEM derived from means of *n* = 3 experiments, superimposed on Beeswarm plots of individual measurements of repeated counts for each of the indicated genotypes. ns, not significant; **p < 0.01, ***p < 0.001 by one-way ANOVA.

To establish the *in vivo* requirement for *tox4*, we generated a null mutant allele of *Drosophila tox4* by imprecise excision of a transposable *P* element inserted in the *tox4* 5′ untranslated region. We identified a strain (referred to as *tox4*^*null*^ hereafter) that carries a 1,180 bp deletion of the *tox4* transcription unit, including the translation start site and most of the coding sequence (Figure 5A). Unlike *PNUTS* null homozygous animals, which die during early larval development,^21^ *tox4*^*null/null*^ animals survived fully to adulthood, although they did not readily thrive. This indicates that zygotic *tox4* is dispensable for normal zygotic development, although early roles may be masked by perdurance of the maternal contribution.

Reduced fecundity of *tox4*^*null*^ animals prompted us to investigate whether fertility was reduced by *tox4* loss of function. Egg production from inbred homozygous *tox*^*null*^ mutant animals was significantly reduced compared with the *w*^*1118*^ control (*p* < 0.0001), laying on average 79.0% ± 2.0% fewer eggs during the monitored period (Figure 5B). *tox4*^*null/null*^ females exhibited the same reduced level of egg production when outbred to *w*^*1118*^ males, and few of the eggs that were laid went on to hatch (11.7% ± 2.8% of *w*^*1118*^ inbred control; *p* ≤ 0.0001, Figure 5C). No larvae emerged from eggs laid by inbred *tox4*^*null/null*^ females. Eggs laid by *w*^*1118*^ females mated to homozygous *tox4*^*null*^ males also showed a severe defect in hatching (*p* < 0.0001), although there were occasionally escapers that progressed to adulthood. This prompted us to examine the requirement of PNUTS binding for *tox4* function in the germline using our genomic GFP-tagged *Drosophila tox4* transgenes. *GFP-tox4*^*wt*^ and -*tox4*^*P216Ter*^ rescued the *tox4* loss-of-function egg laying defect by 74.4% ± 3.6% and 59.2% ± 2.7%, respectively (Figure 5B). *GFP-tox4*^*P216Ter*^ also showed a reduced ability to rescue hatching of embryos laid by *tox4*^*null*^ mothers (57.9% ± 3.5% of controls), compared with *GFP-tox4*^*wt*^ (85.7% ± 3.3% of controls, *p* < 0.0001). There was also a significant difference in the ability of *GFP-tox4*^*wt*^ and *GFP-tox4*^*P216Ter*^ to rescue sterility of *tox4*^*null*^ males (*p* < 0.0001) (Figure 5C). Taken together, these data show that the role of *tox4* in female and male fertility is partially dependent on PNUTS binding.

### A shared role of TOX4, PNUTS, and PP1 results in a nurse cell chromosomal dispersal defect in ovaries

**Figure 6.**
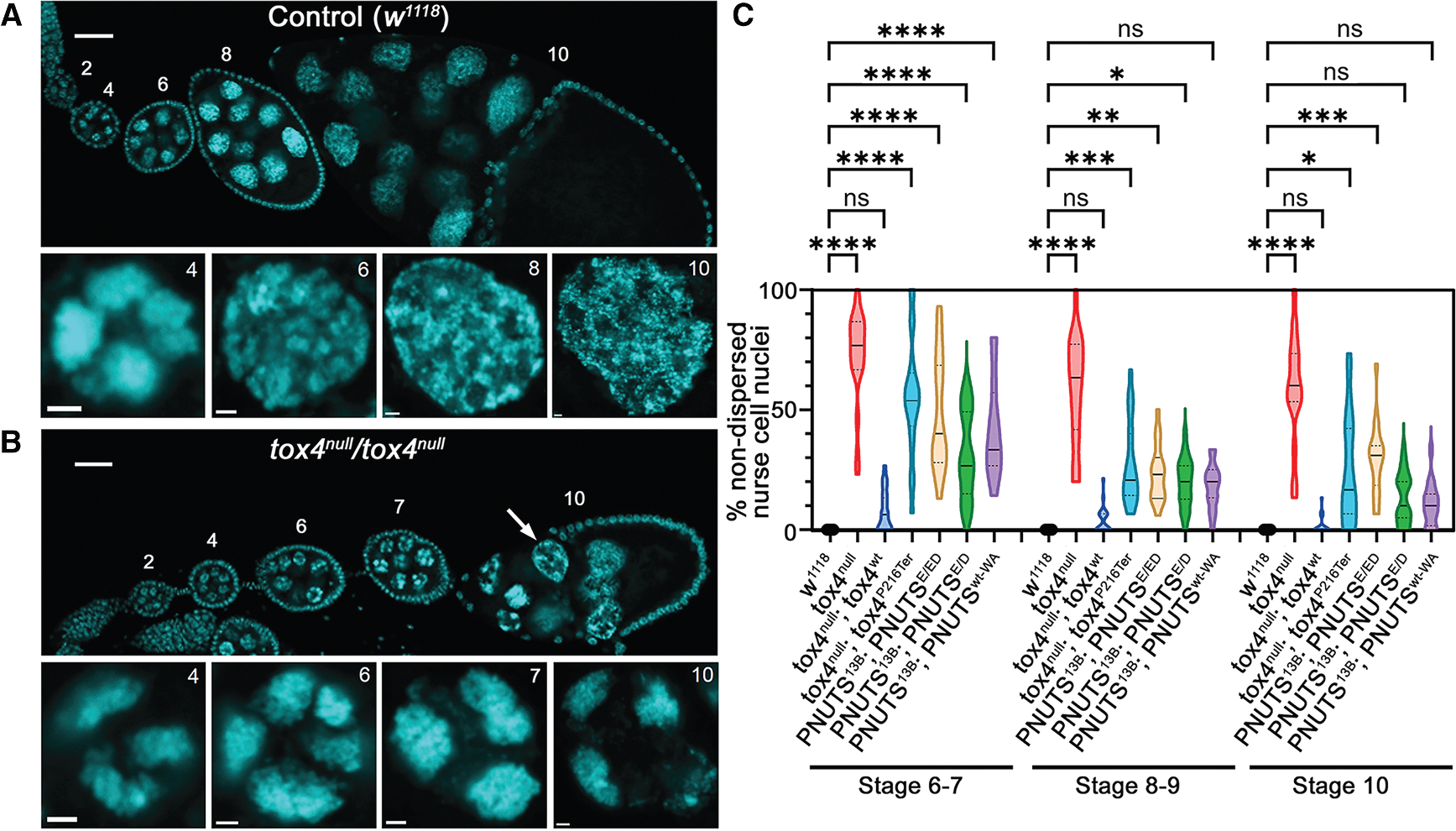
Disruption of PNUTS-PP1 binding, loss of *tox4* function, or loss of PNUTS-Tox4 binding results in chromosome dispersal phenotypes (A and B) Strings of egg chambers of different stages (numbered, up to stage 10) stained with DAPI, which labels both somatic follicular cell nuclei surrounding each chamber and large nurse cell germline nuclei. Lower panels show magnified images of representative nurse cell nuclei from different stages. (A) In *w*^*1118*^ control egg chambers, nurse cell chromosomes disperse throughout the nucleoplasm by stage 6. (B) In contrast, *tox4*^*null*^/*tox4*^*null*^ nurse cell chromosomes fail to disperse, frequently retaining a “five-blob” structure until later stages of development (compare magnified images in A and B). Incomplete dispersal phenotypes were also observed at stage 10, with chromosomes decorating the nuclear periphery without dispersing completely throughout the nucleoplasm (arrow). Scale bars, 40 μm (ovarioles), 2 μm (magnified nuclei). (C) Quantification of non-dispersed nurse cell chromosomes in egg chambers at stage 6–7, 8–9, and 10. Violin plots show mean percentage non-dispersed nurse cell chromosomes/egg chamber for each indicated condition (*n* > 15 egg chambers/stage/genotype). At stage 6–7, all conditions showed significant levels of non-dispersal except *tox4*^*null*^/*tox4*^*null*^ rescued by *GFP-tox4*^*w*t^. *GFP-tox4*^*P216Term*^ failed to substantially rescue dispersal phenotypes at this stage. In all conditions, the extent of non-dispersal decreased with developmental stage. However, significant non-dispersal was still observed for *tox4*^*null*^/*tox4*^*null*^ (with or without *GFP-tox4*^*P216Term*^), and *PNUTS*^*13B*^/^*13B*^ *PNUTS*^*EED*^-GFP at stage 10. ns, not significant; *p < 0.05, **p < 0.01, ***p < 0.001, ****p < 0.0001 by Kruskal-Wallis test.

To understand the functional outcome of the interaction between Tox4 and PNUTS, we analyzed their role in the female germline. Surrounded by a layer of somatic follicle cells, germline nurse cells play an essential role in egg chamber maturation by providing nutrients and support to the developing oocyte. Normally, during the first five endocycles, polytene (64C) nurse cell chromosomes undergo condensin-mediated axial compaction and are visible as a “five-blob” structure up to stage 4 of oogenesis, with each blob representing one of the major chromosomal arms. At the end of the fifth cycle, a mitosis-like phase occurs as the association between somatically paired homologous chromosomes weakens and chromosomes disperse into 32 pairs resulting in a loss of the five-blob structure by stage 6 of oogenesis^45,46^ (Figure 6A). In *tox4*^*null*^ ovaries at stage 6–7, chromosomes failed to fully disperse in nurse cell nuclei (mean non-dispersal 72.7% ± 4.4%), with a majority of nuclei remaining compacted at stage 10 of development (mean non-dispersal 59.0% ± 6.1%, Figure 6B). At stage 6–7, this phenotype was largely rescued by one transgenic copy of *GFP-tox4*^*wt*^ (mean non-dispersal 7.6% ± 2.0%), but incompletely by the PNUTS binding mutant, *GFP-tox4*^*P216Ter*^ (mean non-dispersal 55.5% ± 5.3%). Residual non-dispersal of chromosomes was still evident in 25.2% ± 5.1% of *tox4*^*null*^ *GFP-tox4*^*P216Ter*^ egg chambers at stage 10 (Figures 6C and S7). Temporary persistence of compacted nurse cell chromosomes was also observed in ovaries expressing PNUTS^ED^ or PNUTS^E/ED^ in a *PNUTS*^*13B*^ background (Figures 6C and S7). In PNUTS^E/ED^ egg chambers, where the effect was stronger than PNUTS^ED^, mean non-dispersal was 48.2% ± 4.8% at stage 6–7, decreasing to 29.4% ± 3.3% at stage 10.

Next, we tested if PP1 is important for this nurse cell chromosomal dispersal defect using our inducible *PNUTS*^*wt-flp-W726A*^ transgene. When we specifically induced exchange of PNUTS^wt^ to PNUTS^W726A^ in all ovarian germline cells, we observed persistence of compacted nurse cell chromosomes (mean non-dispersal 41.9% ± 5.3% at stage 6–7, Figures 6C and S6). Thus, considering the similarity in phenotypes observed upon loss of PNUTS-PP1 binding when compared with a loss of *tox4* or of Tox4-PNUTS binding, it is likely that PNUTS, TOX4, and PP1 have a co-operative role in developmentally controlled chromosome reorganization.

### Disruption of PP1 or Tox4 binding to PNUTS results in a common transcriptional response

**Figure 7.**
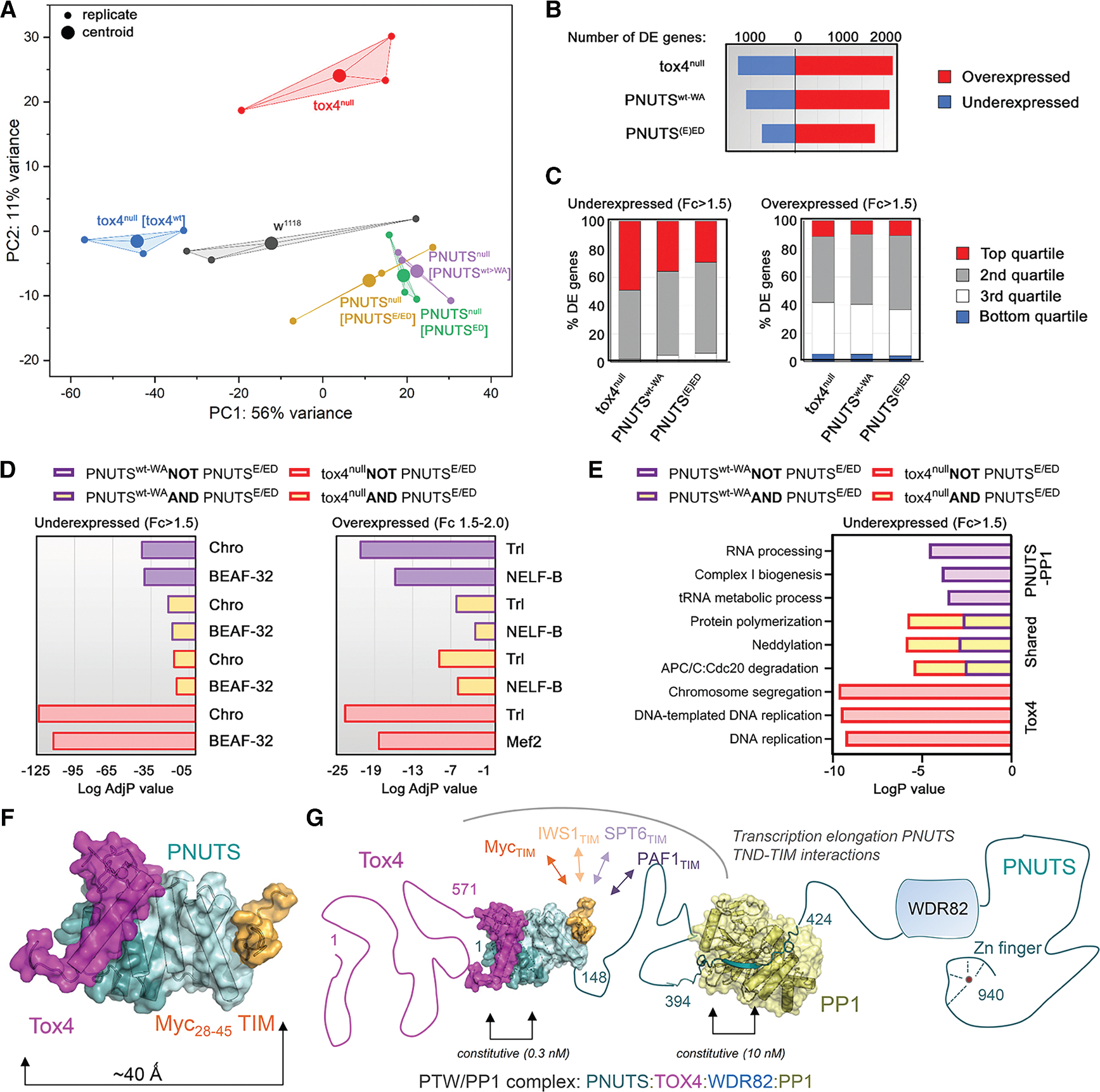
Disruption of PP1 or Tox4 binding to PNUTS induces a common transcriptional signature (A) Principal-component analysis showing common response (PC1) to perturbation of PP1- or Tox4 binding to PNUTS, and divergent response (PC2) to *tox4* loss of function, which together explain approximately 67% of the variance in gene expression. Data points for three independent biological repeats are shown together with the centroid in Euclidian space for each condition: *w*^*1118*^ (gray), *tox4*^*null*^/*tox4*^*null*^ (red), *tox4*^*null*^/*tox4*^*null*^ *GFP-tox4*^*w*t^ (blue), PNUTS^13B^/^13B^ *ovoFLP>PNUTS*^*wt-flp-W726A*^ (purple), *PNUTS*^*13B*^/^*13B*^ PNUTS^ED^ (green), and PNUTS^13B^/^13B^ PNUTS^E/ED^ (yellow). (B) Plot showing number of differentially expressed (DE) genes compared with *w*^*1118*^ control (>1.5-fold over or under-expressed, *p*adj <0.1) for the following conditions: (1) *tox4*^*null*^/^*null*^, (2) *PNUTS*^*13B*^/^*13B*^ *ovoFLP>PNUTS*^*wt-flp-W726A*^, (3) *PNUTS*^*13B*^/^*13B*^ with either PNUTS^ED^ or PNUTS^E/ED^. Overexpressed genes, red bar; underexpressed genes, blue bar. (C) Plot showing percentage of overexpressed and underexpressed genes for each condition as (B) that were found in each quartile of normal expression level derived from read counts in *w*^*1118*^ control. Greater than 90% of underexpressed genes in each condition are in the top two quartiles for normal expression level, whereas >80% of overexpressed genes are more modestly expressed (2nd and 3rd quartiles). (D and E) Gene Ontology (GO) enrichment for DE genes as (B) grouped by Boolean terms, as indicated. (D) Enrichment of transcription factors, for underexpressed (>1.5-fold) and modestly overexpressed (1.5- to 2.0-fold) genes among different conditions compared with *w*^*1118*^ control. The top two statistically significant GO categories (*p*adj < 0.05) for transcription factors (black text)^47^ are shown in each case. (E) Enriched GO terms associated with biological functions among under-expressed genes. (F and G) The PNUTS:PP1:WDR82:TOX4 complex. (F) The PNUTS:Tox4 complex (teal and magenta, respectively) bound to the MYC TIM (PDB: 7LQT; orange). (G) Cartoon illustrating the complex between Tox4 (magenta), PNUTS (teal), PP1 (yellow), WDR82 (blue), and the MYC TIM (orange). Domain interactions for which structures have been determined are shown as cartoon and/or surfaces. Folded interaction partners (WDR82) are shown as a shape, while residues predicted to be IDRs are shown as lines.

To identify molecular signatures associated with loss of Tox4: PNUTS binding, we determined the total mRNA expression profiles from adult ovaries by RNA-seq. Principal-component analysis of the replicates (*n* = 3) showed close agreement between different samples of each line (Figure 7A). Divergent and common responses to loss of *tox4* compared with Tox4-PNUTS binding mutants and controls, agrees with phenotypic analysis indicating that *tox4* possesses both PNUTS-dependent and -independent roles during ovarian development. Strikingly, samples with loss of Tox4-PNUTS binding and loss of PP1-PNUTS binding clustered closely to one another, showing that these conditions share a common response.

Differential gene expression analysis revealed underexpression of a large number of genes (Figure 7B; Table S3), which were in the top quartile of expression in *w*^*1118*^ controls (Figure 7C). Reduction in the level of highly expressed genes enriched in binding sites for the insulator element factors Chromator (Chro), BEAF-32, and CIP190 was common to PNUTS-PP1 binding, PNUTS-Tox4 binding, and *tox4* loss-of-function mutations (Figures 7B–7D). Approximately twice as many genes were overexpressed than were underexpressed for all loss-of-function genotypes compared with the *w*^*1118*^ control (Figure 7B). Human Tox4 has previously been associated with release from proximal RNAPII pausing.^37,38^ Consistent with this, we found that modestly overexpressed (1.5- to 2.0-fold) genes were enriched for pausing factors such as the negative elongation factor, which associates with the GAGA-associated factor/Trl at many paused genes in *Drosophila*^48,49^ (Figure 7D).

To better define common and divergent properties of Tox4 and PNUTS:PP1, we extended a Boolean-logic-based approach to look at enrichment of functional Gene Ontology terms in the gene expression profiles of our different strains (Figure 7E; Table S4). Notably, among the divergent features, we found distinct enrichment of Gene Ontology terms for chromosome segregation and DNA replication among genes underexpressed in *tox4* loss of function, but not in a Tox4 binding mutant of *PNUTS*. We also found unique enrichment of RNA processing and metabolic functions among genes underexpressed in the *PNUTS* PP1 binding, but not Tox4 binding mutant. In contrast, we found shared enrichment of terms for protein polymerization, Neddylation, and APC/C-mediated degradation across conditions, suggesting that these processes are commonly affected by Tox4:PNUTS:PP1.

## DISCUSSION

PNUTS functions as a protein interaction hub that mediates interactions with multiple transcriptional regulators, integrating signals to modulate gene expression. The folded N-terminal domain of PNUTS is an important region of protein-protein interaction, being composed of an N-terminal Tox4BD and a C-terminal TND (TFIIS N-terminal domain) (Figure 2). The TND is a conserved domain recently identified to be enriched in transcription elongation factors such as TFIIS, ELOA, HRP2, and Spn1,^34^ whose function is to interact with short linear motifs present in cognate interacting partners, referred to as TIMs (TND-interacting motifs). Recent work has shown that the conserved PNUTS TND forms a binary interaction module that interacts with IDRs of several transcriptional factors including MYC (Figures 7F and 7G), IWS1, MED13-MED13L, EAF1, EAF2, PAF1, SPT6, and LEO1.^33,34^ Despite its role in binding TIMs, previous data^35^ and this work have shown that the PNUTS_TND_ forms a constitutive complex with Tox4 in cells, suggesting that the Tox4:PNUTS complex must be compatible with dynamic TIM binding. Our data explain how this is achieved. Namely, Tox4 and the transcription factor TIMs bind on opposite surfaces of the PNUTS_TND_ (Figures 7F and 7G).

Our data show that the PNUTS:Tox4 interaction is functionally conserved from flies to mammals. ConSurf analysis^43,44^ shows that the residues that mediate TIM binding, like those that mediate Tox4 binding, are conserved (PNUTS α helix 7 [Leu108, Lys109], α helix 8 [Ala114, Lys115, Lys118, Lys122], and α helix 9 [Ala133, Val137, Trp140, Met141, Ile144]). However, unlike PNUTS_TND_-TIM interactions, which have affinities in the micromolar range and explain the ability of different TIMs to readily displace one another, the PNUTS_Tox4BD_-Tox4 affinity is subnanomolar. This suggests the interaction is constitutive in cells and that this interaction might be leveraged to target the transcription factors that bind the opposite side binding surface of the PNUTS_TND_ to the PNUTS:Tox4:Wdr82 complex (Figure 7G).

The constitutive interaction between Tox4 and PNUTS in the PTW:PP1 complex^35^ implies they have overlapping roles. However, our functional data suggest *tox4* and *PNUTS* also possess independent functions. Most noticeably, *tox4*, unlike *PNUTS*, is dispensable for viability in flies. Consequently, other components of the PNUTS protein interaction hub must be sufficient to mediate *PNUTS*’ essential functions during zygotic development. Moreover, we found that disruption of PNUTS-PP1 binding had a much greater effect on fertility than loss of binding of PNUTS to Tox4, suggesting distinct roles for PNUTS-PP1 during oogenesis. In line with this, we observed enrichment of RNA processing and energy metabolism terms for genes underexpressed in the PNUTS-PP1 binding, but not PNUTS-Tox4 binding mutant strain. These gene signatures may define a separate, Tox-independent role for PNUTS:PP1 that is required for viable egg production. Conversely, the fact that impaired nuclear accumulation in the absence of PNUTS binding does not completely abolish Tox4 function indicates that Tox4 has additional roles beyond its interaction with PNUTS.

Despite being dispensable for viability, *tox4* is required for adult fertility. This requirement is shared by PNUTS:PP1, with loss of Tox4 or PP1 binding to PNUTS leading to similar nurse cell chromosome dispersal defects during oogenesis. Such phenotypes have previously been reported for mutations in transcription factors (E2F1 and DP^50^), a chromodomain protein (Rhino^51^), ribonucleoprotein genes (squid and hrb27C^52^), RNA helicases (P68/*Rm62*^53^), and splicing factors pUf68/hfp, Prp22/pea, snRNP-U1–70K, and U1-snRNA.^54,55^ These data highlight that the Tox4:PNUTS:PP1 complex may regulate the transcriptional programs driving chromosome rearrangements in this context. Nurse-cell chromosome dispersal has been suggested to facilitate rapid synthesis of proteins needed for the remainder of oogenesis from stage 6 onward.^45^ While human oogenesis does not involve chromosome dispersal in the same manner as in the *Drosophila* germline, significant reorganization of chromosome architecture occurs during human oocyte growth and maturation.^56^ These structural changes are associated with transcriptional regulation, contributing to the acquisition of the oocyte’s developmental competence.^57,58^

Previous studies have suggested that chromosome dispersal during *Drosophila* oogenesis involves a transient mitosis-like phase.^45,46^ Underexpressed gene signatures common to PNUTS-Tox4 and PNUTS-PP1 binding mutants were enriched in Neddylation factors and APC/C components that may contribute to the transition between such phases.^59–61^ We also observed underexpression of chromosome segregation genes in *tox4* but not in the *PNUTS* Tox4 binding mutant, perhaps explaining the increased severity of the chromosomal dispersal phenotype and its persistence until later stages of egg chamber development in the *tox4* null strain. Another aspect of the common transcriptional signature in non-Tox4 and non-PP1 binding mutant ovaries was the reduced RNA accumulation for highly expressed genes possessing binding sites for insulator factors, including Chromator and BEAF-32. This may reflect the defects in high-order chromosome structure observed in all the mutants and is consistent with evidence that developmentally controlled chromosomal reorganization, e.g., at the level of topologically associating domains, may be involved in transcriptional reprogramming.^62–64^

The contribution of different components of phosphatase-associated complexes to gene expression and function during development has long been an open question. Our findings demonstrate that recruitment of PP1 and Tox4 to PNUTS is necessary for normal gene expression and chromosomal dispersal during oogenesis. This reveals a mechanism whereby multiple motifs are used combinatorically to tune transcriptional outputs driving developmental transitions.

### Limitations of the study

*Drosophila* was used as a model system to investigate functional consequences of Tox4-PNUTS binding, but whether the PNUTS complex controls germline development in other organisms was not tested. PNUTS is required for nuclear accumulation of Tox4 in *Drosophila* cells. However, whether this is due to changes in the half-life of Tox4, or due to altered nuclear import/export remains to be determined. Finally, RNA-seq experiments identified significant changes to developmental expression profiles in mutant strains. However, which genes are direct targets through which Tox:PNUTS:PP1 control chromosome dispersal remain to be identified.

### RESOURCE AVAILABILITY

#### Lead contact

Further information and requests for resources and reagents should be directed to and will be fulfilled by the lead contact, Daimark Bennett (daimark.bennett@manchester.ac.uk).

#### Materials availability

Plasmids and all unique reagents generated in this study are available from the lead contact with a completed Materials Transfer agreement.

#### Data and code availability

- Atomic coordinates and structure factors have been deposited in the Protein DataBank (PDB: 9CI7). RNA-seq data have been deposited in EMBL-EBI ArrayExpress: E-MTAB-13735.
- This paper does not report original code.
- Any additional information required to reanalyze the data reported in this paper is available from the lead contact (daimark.bennett@manchester.ac.uk) upon request.

## STAR★METHODS

### EXPERIMENTAL MODEL AND STUDY PARTICIPANT DETAILS

*Saccharomyces cerevisiae* strains L40ΔGal4 and HGX13 (Y187 ade2–101: loxP-kanMX-loxP), were used for yeast two hybrid screening as previously reported.^78^

S2R + *Drosophila melanogaster* cells (RRID:CVCL_Z831) used in this study were grown in complete Schneider’s Insect Medium (Sigma) with 10% heat inactivated Fetal Calf Serum (Gibco) and Penicillin-Streptomycin (Invitrogen) at 28°C.

All stocks of *Drosophila melanogaster* were grown and maintained at 18°C and raised at 25°C for experiments on standard fly food media (yeast 50 g/L, glucose 78 g/L, maize 72 g/L, agar 8 g/L, 10% nipagen in EtOH 27 mL/L and propionic acid 3 mL/L).

The following fly lines were used in this study, *w*^*1118*^ (isogenic line), *PNUTS*^*13B*^ (ref. ^21^), *ovo-FLP* (RRID:BDSC_8705), *tox4*^*null*^ (an imprecise excision, this study, of P[EPgy2]CG12104^EY02201^, RRID:BDSC_15089), *GFP-Tox4*^*wt*^ (inserted at *attP40*, this study) *GFP-Tox4*^*P216Ter*^ (inserted at *attP40*, this study), *PNUTS*^*wt*^*-GFP* (inserted at *attP2*, this study), PNUTS^K42E,V44D^*-GFP* (inserted at *attP2*, this study), PNUTS^L10E,K42E,V44D^*-GFP* (inserted at *attP2*, this study) and *PNUTS*^*wt-flp-W726A*^ (inserted at *attP2*, this study).

### METHOD DETAILS

#### Cloning and expression

PNUTS N-terminal domain (5–160, rat) was sub-cloned into pRP1b.^65^ Tox4 C-terminal domain (571–621, human) was sub-cloned into pTHMT vector containing an N-terminal His6-tag followed by maltose binding protein (MBP) and a tobacco etch virus (TEV) protease cleavage site. The PNUTS C48S and Tox4 C601S variants were generated using Quikchange II (Agilent Technologies) and sequence verified. PNUTS and Tox4 constructs were expressed in *E. coli* BL21 (DE3) (Agilent). Cells expressing PNUTS constructs were grown in Luria Broth in the presence of selective antibiotics at 37°C to an OD_600_ of ~0.8, and expression was induced by the addition of 1 mM isopropyl β-D- thiogalactoside (IPTG). Induction proceeded for ~18–20 h at 18°C prior to harvesting by centrifugation at 6,000 ×*g*. Cell pellets were stored at −80°C until purification. For NMR measurements, expression of uniformly ^15^N- and/or ^13^C-labeled PNUTS or Tox4 was achieved by growing cells in M9 minimal media containing 1 g/L ^15^NH4Cl and/or 4 g/L [^13^C]-*D*-glucose as the sole nitrogen and carbon sources, respectively. Tox4 C-terminal domain variants were expressed similarly, except 0.1 mM ZnSO_4_ was added to the Luria Broth or minimal media for proper folding of the protein.

#### Protein purification

PNUTS expressing *E. coli* cell pellets were resuspended in ice-cold lysis buffer (50 mM Tris pH 8.0, 0.5 M NaCl, 5 mM imidazole, 0.1% Triton X-100 containing EDTA-free protease inhibitor tablet [Roche]), lysed by high-pressure cell homogenization (Avestin C3 Emulsiflex) and centrifuged (35,000 ×*g*, 40 min, 4°C). The supernatant was loaded onto a HisTrap HP column (GE Healthcare) pre-equilibrated with Buffer A (50 mM Tris pH 8.0, 500 mM NaCl and 5 mM imidazole) and was eluted using a linear gradient of Buffer B (50 mM Tris pH 8.0, 500 mM NaCl, 500 mM imidazole). Fractions containing the protein were pooled and dialyzed overnight at 4°C (50 mM Tris pH 8.0, 500 mM NaCl, 0.5 mM TCEP) with TEV protease to cleave the His_6_-tag. The cleaved protein was incubated with Ni^2+^-NTA beads (Cytiva) and the flow-through was collected. The protein was concentrated and purified using size exclusion chromatography (SEC; Superdex 75 26/60) pre-equilibrated in NMR Buffer (20 mM MES pH 6, 150 mM NaCl, 5 mM DTT) or crystallization buffer (20 mM Tris pH 8.5, 150 mM NaCl, 0.1 mM ZnSO_4_, 5 mM TCEP). Fractions were pooled, concentrated to designated concentration for experiments or stored at −80°C. Tox4 was purified identically as PNUTS. except Tox4 was incubated with amylose resin to bind cleaved MBP, before being further purified via SEC (Superdex 75 26/60). The Tox4 NMR and crystallization buffers were identical (20 mM Tris pH 8.5, 150 mM NaCl, 0.1 mM ZnSO_4_, 0.5 mM TCEP). To form the PNUTS:Tox4 complex, purified PNUTS and Tox4_C601S_ were mixed at 1: 2 ratio and purified via SEC (Superdex 75 26/60) in 20 mM Tris pH 8.5, 150 mM NaCl, 0.1 mM ZnSO_4_, 0.5 mM TCEP to form 1:1 complex used for crystallization.

#### Crystallization and structure determination

Pooled PNUTS:Tox4 complex in crystallization buffer (20 mM Tris pH 8.5, 150 mM NaCl, 0.1 mM ZnSO_4_, 0.5 mM TCEP) was concentrated to 7 mg/mL. Crystals were formed in 100 mM MES pH 6.5, 1 M LiCl, 15% PEG6K using hanging drop vapor diffusion at room temperature. Crystals were cryo-protected using paraffin oil and immediately flash frozen. Data was collected at SSRL beamline 12.2 at 100 K using a Pilatus 6M PAD detector at two wavelengths, 1.192 Å (remote) and 1.283 Å (inflection, zinc). Data were processed to 2.1 Å using XDS,^66^ Aimless^68^ and Truncate.^67^ The structure was phased using multiple anomalous dispersion (MAD) using the data from both wavelengths and the solve_structure script available at SSRL (http://smb.slac.stanford.edu/facilities/software/MAD_scripts/; script calls Solve and Resolve for phasing and initial model building). Two strong anomalous peaks for zinc were identified, one corresponding to the Tox4 zinc binding site and a second located at a crystal contact. The model of the complex was completed using iterative rounds of refinement in PHENIX and manual building using Coot.^71^ Data collection and refinement details are provided in Table S2.

#### Isothermal titration calorimetry

SEC was used to transfer PNUTS and Tox4 into ITC Buffer (20 mM Tris pH 8.5, 150 mM NaCl, 0.1 mM ZnSO_4_, 0.5 mM TCEP). Purified Tox4 was titrated into PNUTS using an Affinity-ITC at 25°C (TA). Data were analyzed using NITPIC, SEDPHAT and GUSSI.^72,73^

#### NMR spectroscopy

NMR data were collected on Bruker Avance 500, Avance *Neo* 600 and Avance IIIHD 850 MHz spectrometers equipped with TCI HCN *Z*-gradient cryoprobes at 298 K. NMR measurements of PNUTS or Tox4_CTD_ were recorded using ^15^N-labeled protein at a final concentration of 0.1 mM in NMR buffer and 90% H_2_O/10% D_2_O. The PNUTS and Tox4_CTD_ 1:2 complex was formed in Tox4 NMR buffer, and then buffer exchanged to PNUTS NMR buffer (20 mM MES pH 6, 150 mM NaCl, 5 mM DTT) for the NMR measurements. All NMR data were processed using TopSpin 3.5 or 4.05 (Bruker) and analyzed using ccpNMR.^79^

#### Yeast two-hybrid (Y2H) screen

6.1 × 10^7^ *Drosophila* 3^rd^ instar larval cDNA clones were screened in yeast using full-length PNUTS (amino acids 1–1135) protein as ‘bait’ fused to the LexA DNA binding domain in pB27 (a derivative of pBTM116)^80^ by Hybrigenics Inc. Selective medium without tryptophan, leucine and histidine was used to select bait and library plasmids and interactions between the expressed proteins, respectively. 3-aminotriazol (3-AT) an inhibitor of the *HIS3* reporter gene, was used at concentrations up to 50 mM to test binding under increasing stringency in confirmatory assays.^81^ The prey fragment was cloned in frame with the Gal4 Activation Domain (AD) into plasmid pP6, (derived from pGADGH).^82^ The diploid yeast cells were obtained using a mating protocol with L40ΔGal4 (mata) and HGX13 (Y187 ade2–101: loxP-kanMX-loxP, matα) yeast strains.^78^

#### Drosophila DNA cloning and mutagenesis

##### cDNA constructs

The *tox4* coding sequence was cloned from cDNA clone LP01188 (Berkley *Drosophila* Research Project) into pENTR Gateway entry vector and mutagenised to create a truncating Proline to translation stop mutation (Tox4^P216Ter^). Tox4^wt^ and Tox4^P216Ter^ were inserted into N-terminal GFP tagged S2 cell expression vector, pAGW for co-immunoprecipitation (co-IP) and co-localisation studies. PNUTS coding sequence conjugated to a C-terminal Myc tag in the Gateway entry vector pDONR221, was subcloned into pAW for co-IP experiments, and into pARW to create N-terminal RFP fusions for expression in S2 cells. Expression vectors were obtained from the *Drosophila* Genome Resource Center (Indiana, USA). *Genomic constructs*: A 3.3 kb fragment corresponding to the entire *tox4* transcription unit (from 461bp upstream of the ATG to the end of the 3′UTR) was synthesised by GeneArt with the following changes: i) the ORF for eGFP was inserted before the transcription start site to allow visualisation of the expressed protein; ii) an attB site was added at the end of the sequence for ϕC31 site specific recombination^83^; iii) Flanking *Bam*HI and *Not*I restriction sites were added for subcloning into the pCaSpeR4. A variant of this construct was generated that carried Tox4^P216Ter^. For Tox4-binding mutations in PNUTS, we modified an existing 9.1Kb genomic clone (pW8-PNUTS)^21^ to include a 5′ attB site and C-terminal eGFP tag, generating pW8-attB::PNUTS^wt^GFP. The Tox4 binding site was mutagenized via Gibson cloning to generate pW8-attB::PNUTS^K42E, V44D^GFP (PNUTS^ED^) and pW8-attB: PNUTS^L10E,K42E,V44D^GFP (PNUTS^EED^). An inducible PP1 non-binding construct in PNUTS (PNUTS^wt-flp-W726A^) was derived from pW8-attB::PNUTS^wt^GFP to additionally include a duplicated mCherry-tagged 3′ exon harboring a W726A mutation, which significantly decreases the ability of PNUTS to bind to PP1.^21^ The wildtype and W726A-bearing exons, flanked with FRT sites, are irreversibly exchanged by FRT/FLP recombination.

##### Drosophila transgenesis

Site-specific integration into the *Drosophila* genome was performed by the Cambridge Fly Facility, University of Cambridge. pCaSpeR4:tox4^wt^ and pCaSpeR4:tox4^P216Ter^ were inserted into the attP40 landing site. pW8-attB::PNUTS^wt^GFP, pW8-attB::PNUTS^ED^GFP and pW8-attB::PNUTS^EED^GFP were inserted into attP2. *Generation of a Drosophila tox4 null allele*: A null *tox4* allele was generated through imprecise excision of P[EPgy2]CG12104^EY02201^. This was done by crossing the *P* element to P[Δ2–3], a stable source of *P*-transposase,^84,85^ and by screening progeny by PCR to look for deletions in the *tox4* gene. Lesions were confirmed by Sanger sequencing.

##### Egg laying and fertility assay

10 female virgins were crossed to 6 males and allowed to mate for 48 h at 25°C on standard food before transferring onto apple juice agar media. After 24 h, crosses were transferred onto new plates and the first collection of eggs was discarded. Recording of egg counts began with the second plate. The crosses were changed every 24 h and the number of eggs counted. 48 h after egg collections, the number of larvae emerged was counted and the percentage of eggs hatched calculated. Counts from each collection (technical repeats) were used to derive mean measurements for a single experiment. Experiments were performed in triplicate.

#### Transfection of Drosophila S2R + cells

Cells at 50–60% confluency were transiently transfected with Effectene transfection reagent (Qiagen) according to the manufacturer’s protocol and assayed after 48–72 h. Cells were grown in 6-well plastic plates for immunoprecipitation experiments or poly-lysine treated 24-well glass-bottomed Sensoplates (Greiner) for cell imaging.

#### Co-immunoprecipitation and immunoblotting

Transfected S2R + cells were pelleted by centrifugation at 4000 rpm, resuspended in ChromoTek lysis buffer. All buffers were supplemented with 1mM PMSF and 1×Protease Inhibitor Cocktail (Sigma). After incubation on ice for 30 min with pipetting every 5 to 10 min, the lysate was centrifuged at 13,000rpm for 10 min at 4°C and the supernatant transferred to a pre-cooled 1.5mL tube on ice. The supernatant was diluted to 500μL with ice-cold ChromoTek dilution buffer and 20μL of GFP-Trap magnetic beads equilibrated in the same buffer before adding to the lysate. Samples were incubated for 2 h at 4°C on a rotating mixer set at 25 rpm before pelleting the beads using a DiaMag 1.5 magnetic separator (Diagenode) and the buffer removed before washing three times with ice-cold Chromotek wash buffer.

#### Immunostaining and image analysis

S2R+ cells: Cells were live imaged using a Cell Discoverer 7 with Airyscan (Zeiss) equipped with Plan Apochromat 20x/0.95 NA objective, taking a stack of optical sections, each 1μm thick, across every cell. 3D co-localisation and Manders’ coefficient analysis^86^ were carried out using Imaris. Adult *Drosophila* ovaries: After dissection in PBS, tissues were fixed for 30 min in 3.7% paraformaldehyde in PBS at room temperature and then washed three times in PBST (PBS with 0.1% Triton X-100) for 10 min. Staining was performed as previously described.^87^ Ovaries were stained with either Hoechst 33342 or DAPI. After mounting with VectaShield (Vector Laboratories) or ProLong Glass (ThermoFisher) mounting media, fixed tissues were imaged using an LSM710, LSM780, LSM880 (Zeiss) or Nikon A1-R (Nikon Instruments Inc) confocal microscope equipped with 405nm, 488nm, 561nm and 633nm lasers. Tissues were imaged with Plan Apochromat 20x/0.8NA or 40x/1.3NA (oil immersion) objectives. Staging of egg chambers was as previously described.^88^ Measurements of nuclear to cytoplasmic GFP ratio were done in ImageJ, after subtracting mean background signal intensity from the entire image. The mean GFP intensity from at least 4 nuclei/egg chamber was divided by the mean cytoplasmic signal from the corresponding egg chamber to calculate the ratio of nuclear to cytoplasmic GFP for stage 9–10 egg chambers.

#### RNA-sequencing of total RNA following rRNA depletion

Quantity and quality of RNA was quantified using Nanodrop ND-2000 (Thermo Fisher Scientific) and Agilent TapeStation system (Agilent), respectively. RNA-sequencing libraries were constructed using the Stranded Total RNA Prep. Ligation with Ribo-Zero Plus kit (Illumina, Inc.) according to the manufacturer’s protocol with modifications to improve the Ribosomal depletion. Briefly, supplemental oligonucleotide probes (listed in Table S5) were designed corresponding to the following *Drosophila* Ribosomal RNA sequences: 28S, https://www.ebi.ac.uk/ena/browser/view/Non-coding:M21017.1:3288..7232:rRNA; 18S, https://rnacentral.org/rna/URS000030AF9A/7227; 5.8S & 2S, https://www.ebi.ac.uk/ena/browser/view/V00236. Oligonucleotides were purchased lyophilised at 50 pmol/oligo (Integrated DNA Technologies Inc.) and resuspended in RNase-free water to provide a final pool concentration of 1 pmol/oligo. RiboZero Plus reactions were then supplemented (at the ‘Hybridize Probes’ step) with 1 μL of this supplementary oligo pool prior to the addition of 10 μL of the total RNA and continuation of the remaining steps of the RiboZero Plus protocol. 159 bp paired-end reads from biological triplicates for each condition were generated using the NovaSeq6000 system (Illumina). The quality of raw sequence was checked using FastQC. 45–58M reads were generated per sample; ~4% of reads mapped to rRNA after depletion steps described above. Adapters and low-quality bases were removed from the reads using Trimmomatic_0.36.^75^ The trimmer reads were aligned to the *Drosophila* genome version dm6. Counts per gene were calculated using the corresponding annotation with STAR_2.7.7a aligner.^76^ Highly expressed genes were defined as those with normalised read counts in the top two quartiles of the control. Normalisation, principal components analysis (PCA), and differential expression was calculated in DESeq. 2_1.20.0.^74^ Gene ontology and enrichment pathway analysis was performed using FlyenrichR^47^ and Metascape.^77^

### QUANTIFICATION AND STATISTICAL ANALYSIS

For ITC experiments, data were analyzed with a one-site binding model assuming a binding stoichiometry of 1:1 using NITPIC, SEDPHAT and GUSSI. Statistical analyses (mean ± SEM; 3 replicates) of ITC data were completed using Microsoft Excel. For fluorescent imaging, images were captured by Zen (Zeiss) or NIS (Nikon) acquisition software. All images are either single optical sections or maximal intensity projections taken with a confocal microscope. Individual measurements are shown alongside mean values for each condition. Statistical tests (mean ± SEM; ≥3 replicates) were performed in Prism (Graphpad) using a student’s t-test, one-way ANOVA or Kruskal-Wallis test. Unpaired t-tests were used to compare the means of two unmatched groups. One-way ANOVA was used to compare three or more groups, with Tukey correction to compare every mean with every other mean, or Dunnett’s test to compare every mean to the control mean. In cases where data were not found to be normally distributed, we used a non-parametric Kruskal-Wallis test with Dunn’s multiple comparisons to compare the difference in the sum of ranks between conditions.

## Supplementary Material

1

2

SUPPLEMENTAL INFORMATION

Supplemental information can be found online at https://doi.org/10.1016/j.celrep.2025.115693.

## Figures and Tables

**KEY RESOURCES TABLE:** 

REAGENT or RESOURCE	SOURCE	IDENTIFIER

Antibodies		

GFP Polyclonal Antibody	Thermo Fisher Scientific	Cat# A-11122, RRID:AB_221569
c-Myc Monoclonal Antibody (9E10)	Thermo Fisher Scientific	Cat# MA1-980, RRID:AB_558470
Anti-mouse IgG, HRP-linked Antibody	Cell Signaling Technology	Cat# 7076, RRID:AB_330924
Anti-rabbit IgG, HRP-linked Antibody	Cell Signaling Technology	Cat# 7074, RRID:AB_2099233

Bacterial and virus strains		

*E.coli* BL21 (DE3) GOLD expression strain	Agilent	Cat #230132

Chemicals, peptides, and recombinant proteins		

ZnSO_4_	Fisher	Z76-500
PEG6K	Hampton Research	HR2-533
β-D-thiogalactopyranoside (IPTG)	Gold Bio	Cat #367-93-1
Complete Protease inhibitor tablets	Roche	Cat #14696200
Kanamycin	Gold Bio	Cat #K-120-100
Tris (2-carboxyethyl) phosphine hydrochloride (TCEP)	Gold Bio	Cat #TCEP-25
^15^N ammonium chloride 99%	Cambridge Isotope Laboratories	Cat #NLM-465-25
HisTrap HP column	GE Healthcare	17524801
Ni Sepharose 6 Fast flow 1000mL	GE Healthcare	Cat #17-5318-04
HiLoad 26/60 Superdex 75 pg	GE Healthcare	Cat #17-1070-01
Luria Broth	Fisher	BP9722-5
Schneider’s Insect Medium	Gibco	Cat# 21720-024
GFP-Trap magnetic beads	Chromotek	Cat# gtmak
Effectene transfection reagent	Qiagen	Cat# 301425
Fetal Bovine Serum (Heat Inactivated)	Gibco	Cat# A5256801
Penicillin-Streptomycin	Sigma-Aldrich	Cat# P0781
RIPA buffer	Thermo Fisher Scientific	Cat# 89900
SIGMAFAST Protease Inhibitor Tablets	Sigma-Aldrich	Cat# S8820
Laemmli buffer	Sigma-Aldrich	Cat# S3401
ECL Prime Western Blotting Detection Reagent	Cytiva	Cat# RPN2232
DAPI	Thermo Fisher Scientific	Cat# 62248
Hoechst 33342	Thermo Fisher Scientific	Cat# 62249
ProLong Diamond Antifade Mountant	Thermo Fisher Scientific	Cat# P36961

Critical commercial assays		

Stranded Total RNA Prep, Ligation with Ribo-Zero Plus	Illumina	Cat# 20040525

Deposited data		

PDB (Structure of PNUTS:Tox4 complex)	This study	PDBID 9CI7
EMBL-EBI ArrayExpress (RNA sequencing data)	This study	E-MTAB-13735

Oligonucleotides		

See Table S5		

Recombinant DNA		

pRP1b-(rat) PNUTS^5–160^	Peti and Page^65^	N/A
pTHMT-(human) Tox4^571–621^	This study	N/A
pAGW-Drosophila tox4^wt^	This study	N/A
pAGW-Drosophila tox4^P216Ter^	This study	N/A
pAW-Drosophila PNUTS^wt^	This study	N/A
pCaSpeR4-attB, GFP-*Drosophila* tox4^wt^	This study	N/A
pCaSpeR4-attB, GFP-*Drosophila* tox4^P216Ter^	This study	N/A
pw8-attB, *Drosophila* PNUTS^wt^-GFP	This study	N/A
pw8-attB, *Drosophila* PNUTS^K42E,V44D^-GFP	This study	N/A
pw8-attB, *Drosophila* PNUTS^L10E,K42E,V44D^-GFP	This study	N/A
pw8-attB, *Drosophila* pNUTS^wt-flp-W726A^	This study	N/A

Software and algorithms		

Topspin 3.5/4.0.5	Bruker	RRID:SCR_014227
solve_structure script	SSRL	http://smb.slac.stanford.edu/facilities/software/MAD_scripts
XDS	Kabsch et al.^66^	https://www.ccp4.ac.uk/
Truncate	French and Wilson^67^	https://www.ccp4.ac.uk/
Aimless	Evans and Murshudov^68^	RRID:SCR_015747
Solve/Resolve	Terwilliger and Berendzen ^69^	https://solve.lanl.gov/
PHENIX	Zwart et al.^70^	RRID:SCR_014224
COOT	Emsley et al.^71^	RRID:SCR_014222
Pymol	Schrodinger, LLC	RRID:SCR_000305
NITPIC	Scheuermann et al.^72^	https://www.utsouthwestern.edu/labs/mbr/software/
SEDPHAT	Zhao et al.^73^	https://www.utsouthwestern.edu/labs/mbr/software/
GUSSI	Scheuermann et al.^72^	https://www.utsouthwestern.edu/labs/mbr/software/
FIJI (ImageJ)	NIH	RRID:SCR_002285
Zen (v 3.9)	Zeiss	RRID:SCR_013672
Imaris (v 10)	Bitplane/Oxford Instruments	RRID:SCR_007370
Prism (v 10)	Graphpad	RRID:SCR_002798
FastQC	Babraham Bioinformatics	RRID:SCR_014583
DESeq2	Love et al.^74^	RRID:SCR_015687
Trimmomatic	Bolger et al.^75^	RRID:SCR_011848
STAR	Dobin et al.^76^	RRID:SCR_004463
Metascape	Zhou et al.^77^	RRID:SCR_016620
FlyEnrichR	Kuleschov et al.^47^	https://maayanlab.doud/FlyEnrichr

Other		

Cell Discoverer 7 with 20× objective	Zeiss	N/A
LSM880 with 20× objective	Zeiss	N/A
A1R confocal with 20× Plan-Apo objective	Nikon	N/A
24 well Sensoplate	Greiner	Cat# 662892
Coverslips No 1	Scientific Laboratory Supplies	Cat# MIC3110
ChemiDoc MP Imaging System	Biorad	12003154

## Highlights

- The PNUTS:Tox4 structure shows that Tox4 and transcription factors bind distinct surfaces of PNUTS
- *Drosophila* Tox4 is vital for fertility, with some Tox4 fertility effects dependent on PNUTS
- PNUTS-PP1/PNUTS-Tox4 are key for chromosomal dispersal and gene expression during oogenesis
