# Supplementary material for: PNUTS:PP1 recruitment to Tox4 regulates chromosomal dispersal in Drosophila germline development
Source: Cell Rep. Author manuscript; Available in PMC 2025 Jul 21. (PMC12278311; doi:10.1016/j.celrep.2025.115693)
Supplement: 1 [file NIHMS2085660-supplement-1.pdf]

Cell Reports, Volume *44*

Supplemental information

PNUTS:PP1 recruitment to Tox4 regulates chromosomal dispersal in *Drosophila* germline development

Louise Duncalf, Xinru Wang, Abdulrahman A. Aljabri, Amy E. Campbell, Rawan Q. Alharbi, Ian Donaldson, Andrew Hayes, Wolfgang Peti, Rebecca Page, and Daimark Bennett

Table of Contents, this file.

Table S1 Isothermal titration calorimetry (ITC) measurements of PNUTS with TOX4. *Related to Figure 1.*

Table S2 Data collection and refinement statistics. *Related to Figure 2.*

Table S4 Enrichment of Gene Ontology terms. *Related to Figure 7*.

Table S5 Oligonucleotide sequences for rRNA depletion.

Figure S1 2D [^{1}H,^{15}N] HSQC NMR spectrum of ^{15}N-labeled Tox4$_{571-621}$ in the absence (black) and presence (red) of 1 mM Zn^{2+}.

Figure S2 Binding isotherms of PNUTS$_{5-160}$ C48S with Tox4$_{571-621}$ C601S (three replicates shown).

Figure S3 Omit map illustrating the difference density for the bound zinc in the Tox4$_{CTD}$ domain.

Figure S4 Sequence Alignments of PNUTS$_{CTD}$ and Tox4$_{NTD}$.

Figure S5 Gene map of *Drosophila PNUTS* showing position of *PNUTS* transgenes harbouring mutations in Tox4 binding site.

Figure S6 Conditional switching of PNUTS^{wt} to PNUTS^{W726A} in the *Drosophila* germline and associated phenotypes.

Figure S7 Chromosome dispersal phenotypes upon disruption of PNUTS-Tox4 binding.

Table S1: Isothermal titration calorimetry (ITC) measurements of PNUTS with TOX4.

PNUTS variant	Titrant	K_D (nM)	ΔH (kcal/mol)	TΔS (kcal/mol)
PNUTS $_{C48S}$	TOX4 $_{C601S}$	0.3 ± 0.2	-21.1 ± 1.8	-7.9 ± 1.6
$PNUTS_{dead:C48S/L12E/V45D/K43E}$	TOX4 $_{C601S}$	nd	nd	nd

nd, binding not detected

Table S2. Data collection and refinement statistics.

	$PNUTS_{ntd}$:$Tox4_{ctd}$[a] (remote)	$PNUTS_{ntd}$:$Tox4_{ctd}$[a] (inflection)
**PDB**	9CI7	
**Data collection**		
Space group	P 6_1	
Cell dimensions		
a, *b*, *c* (Å)	70.63, 70.63, 97.17	
A, β, γ(°)	90, 90, 120	
Wavelength	1.192	1.283
Resolution (Å)	38.04 – 2.10	38.04 – 2.30
Unique Reflections	15943	12148
R_{merge}	0.06 (0.447)	0.07 (0.372)
Mean I /σI	9.0 (1.7)	8.7 (2.1)
Completeness (%)	96.6 (83.1)	98.8 (96.0)
Multiplicity	2.8 (2.7)	2.8 (2.6)
$CC_{1/2}$	0.973 (0.735)	0.972 (0.806)
**Refinement**		
Resolution (Å)	35.12 - 2.10 (2.23 - 2.10)	
No. reflections	15920	
R_{work} / R_{free}	0.19 (0.25)/0.22(0.29)	
No. atoms		
Protein	1600	
Ligand	7	
Water	128	
B-factors		
Protein	46.7	
Ligand	50.2	
Water	48.3	
RMS deviations		
Bond lengths (Å)	0.001	
Bond angles (°)	0.34	
Ramachandran		
Outliers (%)	0.0	
Allowed (%)	1.5	
Favored (%)	98.5	
Clashscore	0.0	

[a]Data was collected from a single crystal

*Values in parentheses are for highest-resolution shell

Table S4: Enrichment of Gene Ontology terms. *Related to Figure 7*.

LogP_PNUTS^{wt-WA} **NOT** PNUTS$^{E/ED}$	LogP_PNUTS^{wt-WA} **AND** PNUTS$^{E/ED}$	LogP_tox4^{null} **NOT** PNUTS$^{E/ED}$	LogP_tox4^{null} **AND** PNUTS$^{E/ED}$	GO	Description	Hits	Enrichment	#Total Gene In Library	#Gene In GO	#Gene In GO And Hit List	%InGO	STDV % In GO
0.00	0.00	-9.68	0.00	GO:0007059	chromosome segregation	Klp3A, Nek2, feo, Kmn1, nod, Ndc80, Mer, Wee1, Nuf2, borr, aurB, dgt2, Orc5, Incenp, dgt5, cid, mars, tum, SMC2, Lis-1, tef, thr, prod, dgt3, msd1, msd5, Mis12, Klp67A, CG3437, polo, mgr, Orc2, Mps1, Mink, dgt6, Slbp, sub, c(3)G, Zw10	3.08	12585	193	39	4.72	0.74
0.00	0.00	-9.54	0.00	GO:0006261	DNA-templated DNA replication	Chrac-16, CG8142, CG3430, CG6443, RfC38, Orc5, PolD3, E2f2, Orc1, Orc6, Ctf4, nopo, PolE4, DNAlig1, Orc4, Psf1, RfC4, hd, Mcm5, Orc2, mtSSB, Sld5, spn-A, Prim2, dup	4.27	12585	89	25	3.02	0.60
0.00	0.00	-9.30	0.00	GO:0006260	DNA replication	Chrac-16, CG8142, CG13690, CG3430, CG6443, RfC38, Orc5, PolD3, E2f2, Orc1, Orc6, Ctf4, nopo, PolE4, DNAlig1, Orc4, Psf1, RfC4, hd, Mcm5, Orc2, mtSSB, Sld5, spn-A, Prim2, dup	4.04	12585	98	26	3.14	0.61
0.00	0.00	-7.60	0.00	GO:0007051	spindle organization	Klp3A, feo, nod, Wee1, aurB, dgt2, Orc5, Incenp, dgt5, cid, mars, tum, Lis-1, dgt3, msd1, msd5, Klp67A, CG3437, alphaTub67C, spd-2, polo, mgr, aurA, Orc2, dgt6, sub, ghi, Tap42	3.23	12585	132	28	3.39	0.63
0.00	-2.73	0.00	-3.10	GO:0051258	protein polymerization	dgt4, Grip128, gammaTub23C, CCDC53, Grip75, dgt2, dgt5, dgt3, CG3085, wac, msd1, msd5, polo, Sas-4, SelR, dgt6, sub	2.90	12585	47	17	1.08	0.26
0.00	-2.95	0.00	-2.96	R-DME-8951664	Neddylation	CG11700, Fbxl4, Charon, Uba3, CG3894, CG7168, CG1523, CG42233, l(2)dtl, Ubi-p5E	3.22	12585	131	10	3.36	1.04
0.00	-2.61	0.00	-2.87	R-DME-174048	APC/C:Cdc20 mediated degradation of Cyclin B	CG11700, APC4, Ubc2, Ubi-p5E	8.04	12585	21	4	1.34	0.67
0.00	-2.46	0.00	-2.72	R-DME-179409	APC-Cdc20 mediated degradation of Nek2A	CG11700, APC7, Nek2, APC4, Ubc2, fzy, mad2, Cdc27, Ubi-p5E	3.14	12585	23	9	0.57	0.19
-4.61	0.00	0.00	0.00	GO:0006396	RNA processing	MTPAP, CG14805, Rtca, CG3071, Pop1, wuho, Bx42, CG2124, Dus2, CG9123, CG8097, Rrp45, Ssu72, Dus1, CG4291, IntS14, Tgt, snRNP-U1-70K, Srp54, aub, zuc, CG4935, Rpp25, Ctu1, Cdc2rk, Lsm10, cuff, CG11808, Rrp42, CG15084, tapas, CG9752, CG7974, CG2021, CG11586, ldbr, CG6833, IntS9, Smn, Pop5, CG17187, Dph3, CG5641, CG3817, Mettl4, LSm3, CG14543, Slbp, pasha, snoRNA:Psi28S-2876, snoRNA:Me18S-A1576, snRNA:U4:38AB, snoRNA:Psi18S-1377c, snoRNA:Psi28S-3327a, snoRNA:Psi28S-3316b, snoRNA:Psi28S-3327b	1.76	12585	707	56	9.88	1.25
-3.90	0.00	0.00	0.00	R-DME-6799198	Complex I biogenesis	ND-B18, ND-24, ND-B14.5B, CG15653, ND-30, ND-39, ND-PDSW, ND-AGGG, Hsc20, CG34229	4.11	12585	54	10	1.76	0.55
-3.57	0.00	0.00	0.00	GO:0006399	tRNA metabolic process	CG10802, Pop1, wuho, Dus2, CG8097, Rrp45, Dus1, Tgt, AsnRS, Rpp25, Ctu1, Rrp42, CG9752, Tbp, Pop5, Dph3, beta-PheRS, Polr2L	2.53	12585	158	18	3.17	0.74
-3.54	0.00	0.00	0.00	R-DME-611105	Respiratory electron transport	fh, ND-B18, ND-24, ND-B14.5B, CG15653, ND-30, ND-39, COX8, COX7AL, ND-PDSW, RFeSP, ND-AGGG, UQCR-11, Hsc20, CG34229	2.80	12585	119	15	2.65	0.67

Table S5: Oligonucleotide sequences for *Drosophila* rRNA depletion. Anti-sense probe designs 50 nt in length are based on the following fasta file sequences, M21017.1:3288..7232:rRNA, V00236, URS000030AF9A_7227.

Drosophila rRNA (2S,5.8S,18S,28S) #1	CTATCAATCTGTCTTACACACTTATGTTCGACCTGGTAAGTTTTCCCGTG
Drosophila rRNA (2S,5.8S,18S,28S) #2	TTCAACTTTCCCTCACGGTACTTGTTTACTATCGGTCTCATGGTTATATT
Drosophila rRNA (2S,5.8S,18S,28S) #3	TTTACCACCCACTTAGTGCTGCACTATCAAGCAACACGACTCTTTGGAAA
Drosophila rRNA (2S,5.8S,18S,28S) #4	TGGTAGTCCTAGATACTACCATCAAAAGTTGATAGGGCAGACATTTGAAA
Drosophila rRNA (2S,5.8S,18S,28S) #5	CTTTTATATGTAACTAGCGCGGCATCAGGTGATCGAAGATCCTCCCAATT
Drosophila rRNA (2S,5.8S,18S,28S) #6	TACTATCCACCAAGATCTGTACCAATGGCAGCTCCATGCAGGCTTACGCC
Drosophila rRNA (2S,5.8S,18S,28S) #7	TGACTTCAACCTGATCAAGTATAGTTCACCATCTTTCGGGTCACAGCATA
Drosophila rRNA (2S,5.8S,18S,28S) #8	TCCCCACCATAAATTTTCAAGGTCCGAGGAGAAAATATCGACACAACAGT
Drosophila rRNA (2S,5.8S,18S,28S) #9	ATGTTCATGTGTCCTGCAGTTCACACGATGACGCACAGTTTGCTGCGTTC
Drosophila rRNA (2S,5.8S,18S,28S) #10	ACTTCCCCCGGAGCCCAAAAAGCTTTGGTTTCCCGGAAGCGACTGAGAGA
Drosophila rRNA (2S,5.8S,18S,28S) #11	GTCCTTGAATTGGATCATACCTGAGTAATTGGAGTTATACCAAATTTTCA
Drosophila rRNA (2S,5.8S,18S,28S) #12	ATTAACGTTATACGGGCCTCATTTAAGAAGGACTTAAATCGTTAATTTCT
Drosophila rRNA (2S,5.8S,18S,28S) #13	TTTATCAAATTAATAACATTTATTCTGTGTTAAAATGCAAGCAATTTAAT
Drosophila rRNA (2S,5.8S,18S,28S) #14	AAAACTCTTCCGATATCTCTCGACGGCTTCTTTATGGTCGTTCCTGTTGC
Drosophila rRNA (2S,5.8S,18S,28S) #15	AGGTTTATATCCCAATAACTTGCACATATGTTAGACTCCTTGGTCCGTGT
Drosophila rRNA (2S,5.8S,18S,28S) #16	CGACGGTCCAAGAATTTCACCTCTCGCGTCGTAATACTAATGCCCCCAAA
Drosophila rRNA (2S,5.8S,18S,28S) #17	CAACCACTGTTCACACGAAACCCTTCTCCACTTCAGTCCTCCAAGGCTCT
Drosophila rRNA (2S,5.8S,18S,28S) #18	TTGTTAAAGGATTTAAAGTGTACTCATTCCAATTACAGGGCCTCGGATAT
Drosophila rRNA (2S,5.8S,18S,28S) #19	TAATAAGAACTAATTGGTTTAACCCAAATAGTATTCTTAAAAATTTTAGC
Drosophila rRNA (2S,5.8S,18S,28S) #20	AGGAAATACACGTTGATACTTTCATTGTAGCGCGCGTGCAGCCCAGGACA
Drosophila rRNA (2S,5.8S,18S,28S) #21	AACAGTTTCAGTTCACAATCCCAAGCATGAAAGTGGTTCAGCGGTTTACC
Drosophila rRNA (2S,5.8S,18S,28S) #22	ACACCTTGCCTGTTACAAAAGTCGTTTACAATTGATTCTAGGCTTTGTCA
Drosophila rRNA (2S,5.8S,18S,28S) #23	TATAATGGGACAAACCAACAGGTACGGCTCCACTTACATAAACACATTCA
Drosophila rRNA (2S,5.8S,18S,28S) #24	TGTGTTAATTAGCTATAAATAGCTAAAAAACTAATCCCATTATTAGTCAA
Drosophila rRNA (2S,5.8S,18S,28S) #25	TACATTGGCATCACATCCATTGTCGTTTATAAAGTAAATTATAAACTTTA
Drosophila rRNA (2S,5.8S,18S,28S) #26	ATTACCTCTTGATCTGAAAACCAATGAAAGCAGAACAGAGGTCTTATTTC
Drosophila rRNA (2S,5.8S,18S,28S) #27	AGACAGTCGGATTCCCCAAGTCCGTGCCAGTTCTGAATTGATTGTTAATT
Drosophila rRNA (2S,5.8S,18S,28S) #28	AAGTATTTAATCACATATAAGAACTCCACCGGTAATACGCTTACATACAT

Drosophila rRNA (2S,5.8S,18S,28S) #29	TTCCAGTTAGAGGCATAAATAATATAAATATACATTATACATAACTATAT
Drosophila rRNA (2S,5.8S,18S,28S) #30	TCTTCATATCAAGAAAGTTAAGGTTCTTACCCATTTAAAGTTTGAGAATA
Drosophila rRNA (2S,5.8S,18S,28S) #31	TGTTAAAAACTCTTTAAACCAAAAGGATCGATAGGCCGAGCTTTTGCTGT
Drosophila rRNA (2S,5.8S,18S,28S) #32	CTTCCTCTAAATAATCAAGTTCGGTCAACTTTTGCGAAACAACCGTAACA
Drosophila rRNA (2S,5.8S,18S,28S) #33	CTTTAGCGGTAATGTATAGGTATACAACTTAAGCGCCATCCATTTTAAGG
Drosophila rRNA (2S,5.8S,18S,28S) #34	TTTTCGCTCGCCGCTACTAAGAAAATCCTTGTTAGTTTCTTTTCCTCCCC
Drosophila rRNA (2S,5.8S,18S,28S) #35	TCGAATCATCAAGCAAAGGATAAGCTTCAGTGGATCGCAGTATGGCAGCT
Drosophila rRNA (2S,5.8S,18S,28S) #36	CAAGACCATACGATCTGCATGTTATCTAGAGTTCAACCAATATAACGATC
Drosophila rRNA (2S,5.8S,18S,28S) #37	ACGTCGCTATGAACGCTTGGCCGCCACAAGCCAGTTATCCCTATGGTAAC
Drosophila rRNA (2S,5.8S,18S,28S) #38	ACACCGAGATCAAGTCAGCATTTGCCCTTTTGCTCTATGTGTGGTTTCTG
Drosophila rRNA (2S,5.8S,18S,28S) #39	GAAGGTATCCTGAATCTTTCGCATTGTTAATCATACAAGTGCATATAATA
Drosophila rRNA (2S,5.8S,18S,28S) #40	CTCTAACTTTCGTTCTTGATTAATGAAAACATCTTTGGCAAATGCTTTCG
Drosophila rRNA (2S,5.8S,18S,28S) #41	CGTTATAAAAAATTTATAAGCACTTAATTAGCATAGTCTTACAACCCTCA
Drosophila rRNA (2S,5.8S,18S,28S) #42	GTTATATTTTATGATAAATTTGGTATATGCTAATAGATTACAATGTCCTT
Drosophila rRNA (2S,5.8S,18S,28S) #43	CCTTGGGACACCTCCGTTATTATTTGAGAGATGTACCGCCCCAGTCAAAC
Drosophila rRNA (2S,5.8S,18S,28S) #44	AGCAGCACTATAAAATTAATTAAAGTACATAACAGCATGGACTGCGATAT
Drosophila rRNA (2S,5.8S,18S,28S) #45	ACACCCAATTGCTAGCTGGCATCGTTTATGGTTAGAACTAGGGCGGTATC
Drosophila rRNA (2S,5.8S,18S,28S) #46	CTAGAGACTCTTCACCTTGGAGACCAGCTGCGGATATTGGTACGGCCTGT
Drosophila rRNA (2S,5.8S,18S,28S) #47	AGCACTGGGCAGAAATCACATTGTGTCAACACCCGCTAGGGCCATCACAA
Drosophila rRNA (2S,5.8S,18S,28S) #48	CCAAACCGAGGTCTAATATCTCCCACTTATTCTACACCTCTTATGTCTCC
Drosophila rRNA (2S,5.8S,18S,28S) #49	AGGCATAATCCAACGGACGTAGCGTCATACCACTGTTCGCTCGAACAAGT
Drosophila rRNA (2S,5.8S,18S,28S) #50	CAGGTTCATCGGGCTTAACCTCTAAGCAGTTTCACGTACTGTTTAACTCT
Drosophila rRNA (2S,5.8S,18S,28S) #51	GATAATTATGCCATTATATAATTCCGAAAAATTAACGCACTGTAATCATA
Drosophila rRNA (2S,5.8S,18S,28S) #52	AGGGCAGGGACGTAATCAATGCGAGTTAATGACTCACACTTACTGGGAAT
Drosophila rRNA (2S,5.8S,18S,28S) #53	TATACTCAATTCTGACAATCGATTTGCACGTCAGAACTGTTTCGGTCTTC
Drosophila rRNA (2S,5.8S,18S,28S) #54	AGGTGAGTTGTTACACACTCCTTAGCGGATTTCGACTTCCATGATCACCG
Drosophila rRNA (2S,5.8S,18S,28S) #55	GCCGAGTGATCCACCGCTTAGAGTTTTATAATTCATTTTTATATAATGTC
Drosophila rRNA (2S,5.8S,18S,28S) #56	AGAGAGTCATAGTTACTCCCGCCGTTGACCCGCGCTTACTTGAATTTCTT
Drosophila rRNA (2S,5.8S,18S,28S) #57	GCCCATATTTAATAACAAACGGATACTCAACAGGTTACGGAATTGGAACC
Drosophila rRNA (2S,5.8S,18S,28S) #58	CATCCCACAGCGCCAGTTCTGCTTACCAAAAGTGGCCCACTGGGACATTT
Drosophila rRNA (2S,5.8S,18S,28S) #59	GAATATTCAGGCATTTGAAGCCTGCTTTAAGCACTCTAATTTGTTCAAAG

Drosophila rRNA (2S,5.8S,18S,28S) #60	CAAAATTATGTTTTACTTGAAAATTTTCGCTTTCGCCTTGAACTTAGGAC
Drosophila rRNA (2S,5.8S,18S,28S) #61	CGTACCTGCGGGTTCCTCTCGTACTACGCAGGAATGCTGTCGCAACAACG
Drosophila rRNA (2S,5.8S,18S,28S) #62	AAGCTTGCATCAAAACCCAATACCATAAGATATAATAAATATATCCGTAT
Drosophila rRNA (2S,5.8S,18S,28S) #63	GACCTGTTATTGCTCAATCTCATTATTGCTAGACGCAATTTGTCCATTTA
Drosophila rRNA (2S,5.8S,18S,28S) #64	TTTAGCCTAATAAAAGCACACGTCCCATAAGGTTCATGTTTTAATTGCAT
Drosophila rRNA (2S,5.8S,18S,28S) #65	TTACTGCCAACATGAATGAAGGCTACATAAGCTTCAGCACCATAATCCTG
Drosophila rRNA (2S,5.8S,18S,28S) #66	ATTTTTCGTCACTACCTCCCCGAGCTGGGAGTGGGTAATTTACGCGCCTG
Drosophila rRNA (2S,5.8S,18S,28S) #67	ACAATAACACTCGTTTAAGAGCACTAATGCAGGTTTTTAAATAGGAGGAA
Drosophila rRNA (2S,5.8S,18S,28S) #68	TACCATTGTACCTTCCTACTCACTAAAGTTTCAAAATTTATATCACAAGT
Drosophila rRNA (2S,5.8S,18S,28S) #69	CTAGCCCATCTACCATATCTCTCTGCGAAAGACTTCCATGGTAGTACGGC
Drosophila rRNA (2S,5.8S,18S,28S) #70	CAGCTATCCTGAGGGAAACTTCGGAAGGAACCAGCTACTAGATGGTTCGA
Drosophila rRNA (2S,5.8S,18S,28S) #71	AATGCATCGTTTTATTAAAGAATTTGTTTGCGATTATATAACAAACTCGT
Drosophila rRNA (2S,5.8S,18S,28S) #72	CAACCAACGCCTTTCATGGTATCTGCATGAGTTGTTAATTTGGGCACGTA
Drosophila rRNA (2S,5.8S,18S,28S) #73	GCGGTTTCACTTTTAATTCGTGTGTACTTAGACATGCATGGCTTAATCTT
Drosophila rRNA (2S,5.8S,18S,28S) #74	TTGGCTACGTAACTAAACTATCCGGGGAACAAGTAACTAACATAAATGCT
Drosophila rRNA (2S,5.8S,18S,28S) #75	TTACCACAGTTATCCAAGTAACTGTTAACGATCTAAGGAACCATAACTGA
Drosophila rRNA (2S,5.8S,18S,28S) #76	CCAGAACGAGCACATAAACCATGTTATTGTTTCCCAATCAAGGCCGACTA
Drosophila rRNA (2S,5.8S,18S,28S) #77	ATACAATGCAAATTGCCCCTTATTTATCATTGCAGTCCAGCACGGATACG
Drosophila rRNA (2S,5.8S,18S,28S) #78	GACCCTAAGGCCTCTAATCATTCGCTTTACCAGATAAGATTATTTTATAT
Drosophila rRNA (2S,5.8S,18S,28S) #79	ACACTATTCTCATAATATTATTTAAATATTACAATTTTAATGATGAATTT
Drosophila rRNA (2S,5.8S,18S,28S) #80	ACCACAATTGTAAGTTGTACTACCCGTATGAAGCACAAGTTCAACTACGA
Drosophila rRNA (2S,5.8S,18S,28S) #81	CCAATCCTTATCCCGAAGTTACGGATCTAATTTGCCGACTTCCCTTACCT
Drosophila rRNA (2S,5.8S,18S,28S) #82	ATACACGTTCCATTTAATCAAGTAAGTAAGGAAACAATAAGAGTAGTGGT
Drosophila rRNA (2S,5.8S,18S,28S) #83	ACGCTCCATACACTGCATCTCACATTTGCCATATAGACAAAGTGACTTAG

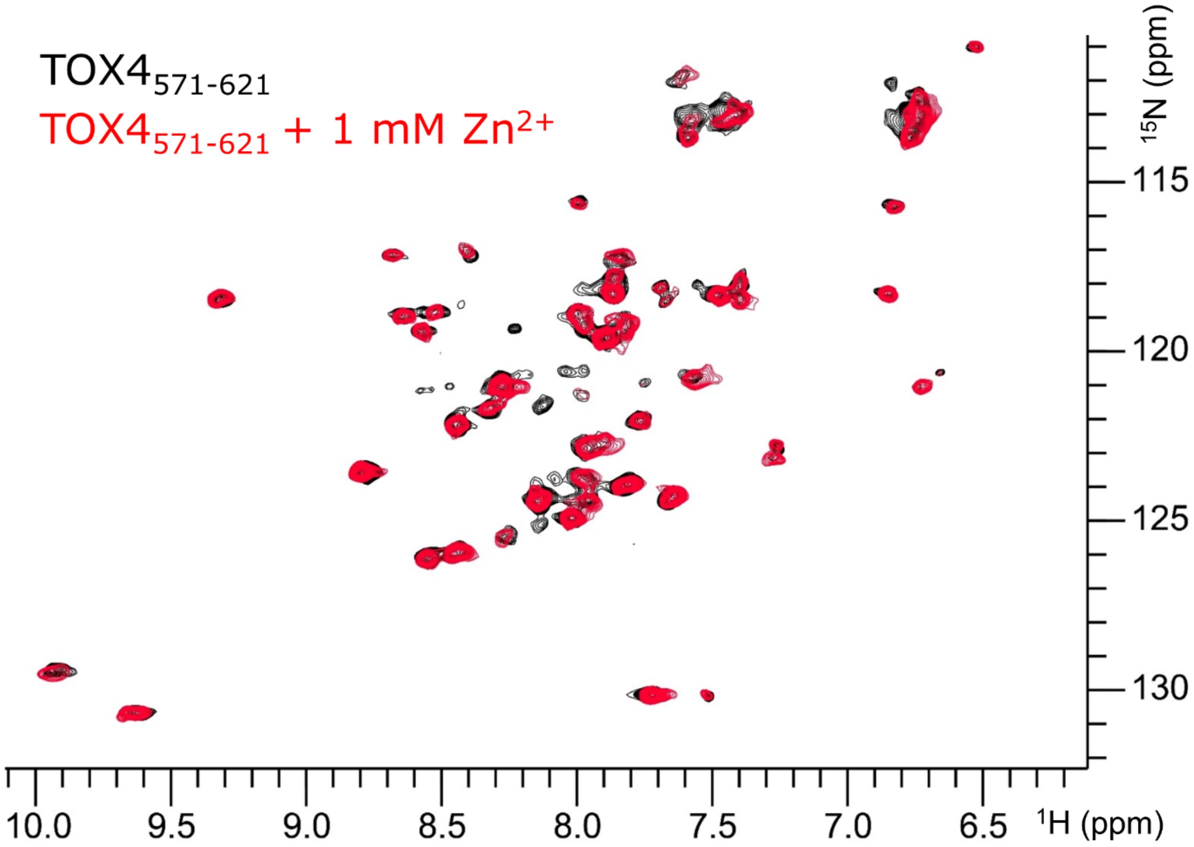

Figure S1. 2D [^{1}H,^{15}N] HSQC NMR spectrum of ^{15}N-labeled Tox4$_{571\text{-}621}$ in the absence (black) and presence (red) of 1 mM Zn^{2+}.

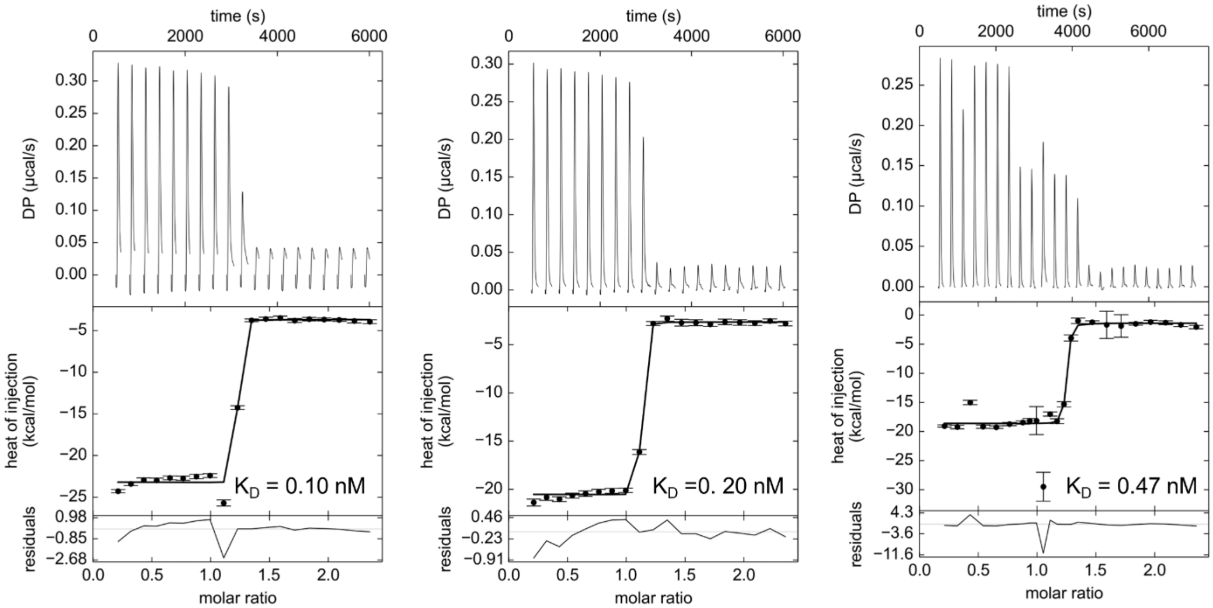

Figure S2. Binding isotherms of $PNUTS_{5\text{-}160}$ C48S with $Tox4_{571\text{-}621}$ C601S (three replicates shown).

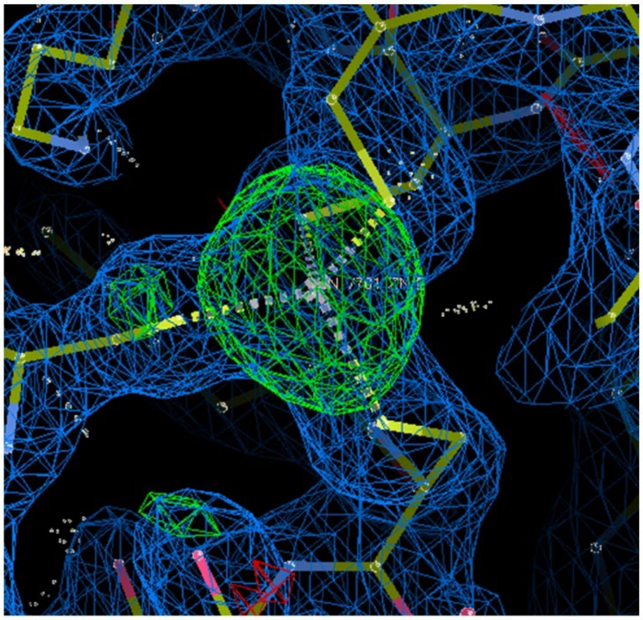

Figure S3. Omit map illustrating the difference density for the bound zinc in the Tox4$_{CTD}$ domain (2F_o-F_c, blue; positive F_o-F_c, green).

PNUTS$_{NTD}$

```
human        5 --PIDPKELLKGLDSFLTRDGEVKSVDGIAKIFSLM-KEARKMVSRCTYLNIILQTRAPE    61
rat          5 --PIDPKELLKGLDSFLNRDGEVKSVDGISKIFSLM-KEARKMVSRCTYLNILLQTRSPE    61
fly          1 MPRIVPLQLLRCLRVLLDNNGGILSAVEVKRISGLMAKYSKKLVSKCVYVQILK-STKTE    59
                  * * :**: *  :* .:* : *.  : :* .** * ::*:**:*.*::*:  :   *

human       62 VLVKFIDVGGYKLLNSWLTYSKTTNNIPLLQQILLTLQHLPLTVDHLKQNNTAKLVKQLS   121
rat         62 ILVKFIDVGGYKLLNNWLTYSKTTNNIPLLQQILLTLQHLPLTVDHLKQNNTAKLVKQLS   121
fly         60 LLGDFMAVGGWSLVYTWLNDAIRAMNWPLVQEILELLLLSPVDVNRLKINSAPILVKGLC   119
               :* .*: ***:.*: .**. :  : * **:*:**  *   *: *::** *.:  *** *.

human      122 KSSEDEELRKLASVLVSDWMAVIRSQSSTQPAEKDKKKR   160
rat        122 KSSEDEELRKLASVLVSDWMAVIRSQSSTQPAEKDKKKR   160
fly        120 KDGGNEGVRILAKRLVEQWLKIVTENTSMTIQGAAPHIA   158
               *.. :* :* **. **.:*: :: .::*        :
```

Tox4$_{CTD}$

```
human      571 RCVRSGCENPPIVSKDWDNEYCSNECVVKHCRDVFLAWVASRNSNTVVFVK   621
fly        207 KCTREQCNKPAIINPDWEDEYCSNECVVIHCRNVFNHWVISMNS-------   250
               :*.*. *::* *:. **::********* ***:**  ** * **
```

Residues at the Tox4$_{CTD}$:PNUTS$_{NTD}$ interface

Residues mutated as described in Figure 4

Figure S4. Sequence Alignments of PNUTS$_{CTD}$ (top) and Tox4$_{NTD}$ (bottom). The isoforms used for the crystal structure of the Tox4$_{CTD}$:PNUTS$_{NTD}$ complex were human (Tox4, C601S) and rat (PNUTS, C48S). The sequence identity between PNUTS$_{NTD}$ human/rat is 96%; the 6 residues that differ between them are bolded in the sequence above. Residues used for mutagenesis to disrupt TOX4/PNUTS binding are indicated by arrows, demonstrating these residues are conserved between human/rat/fly. Residues that comprise the Tox4$_{CTD}$:PNUTS$_{NTD}$ interface (as determined by calculating the change in the solvent accessible surface area upon binding) are highlighted in yellow.

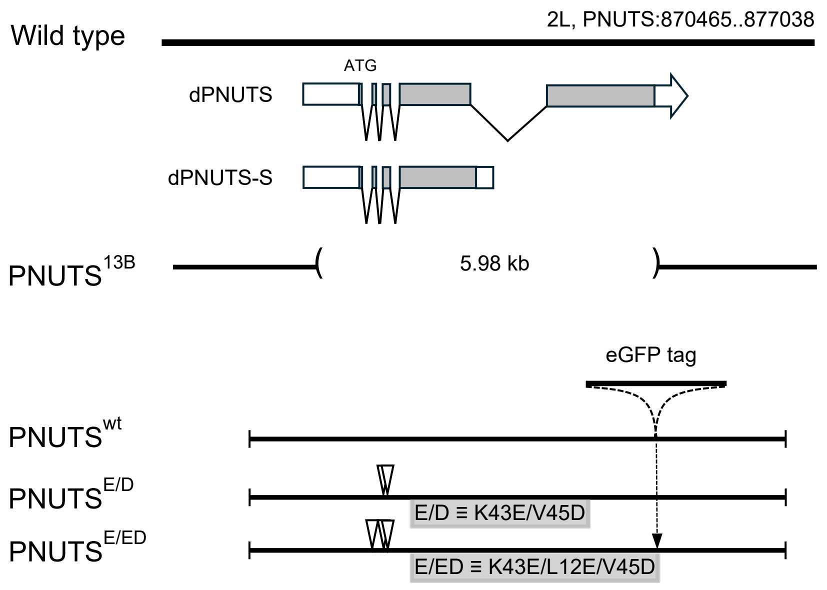

Figure S5. Gene map of *Drosophila PNUTS* and *PNUTS* transgenes harbouring mutations in Tox4 binding site. Genomic region and intron-exon structure of *Drosophila PNUTS* transcription unit. Grey shading represents coding regions, unfilled boxes represent untranslated regions with arrows indicating direction of transcription. *PNUTS*^{13B} harbours a deletion removing all the coding sequence and some untranslated region of *PNUTS*, as previously reported (Ciurciu et al., 2013). Illustration below shows the position genomic transgenes carrying either wildtype or mutant *PNUTS* tagged with GFP.

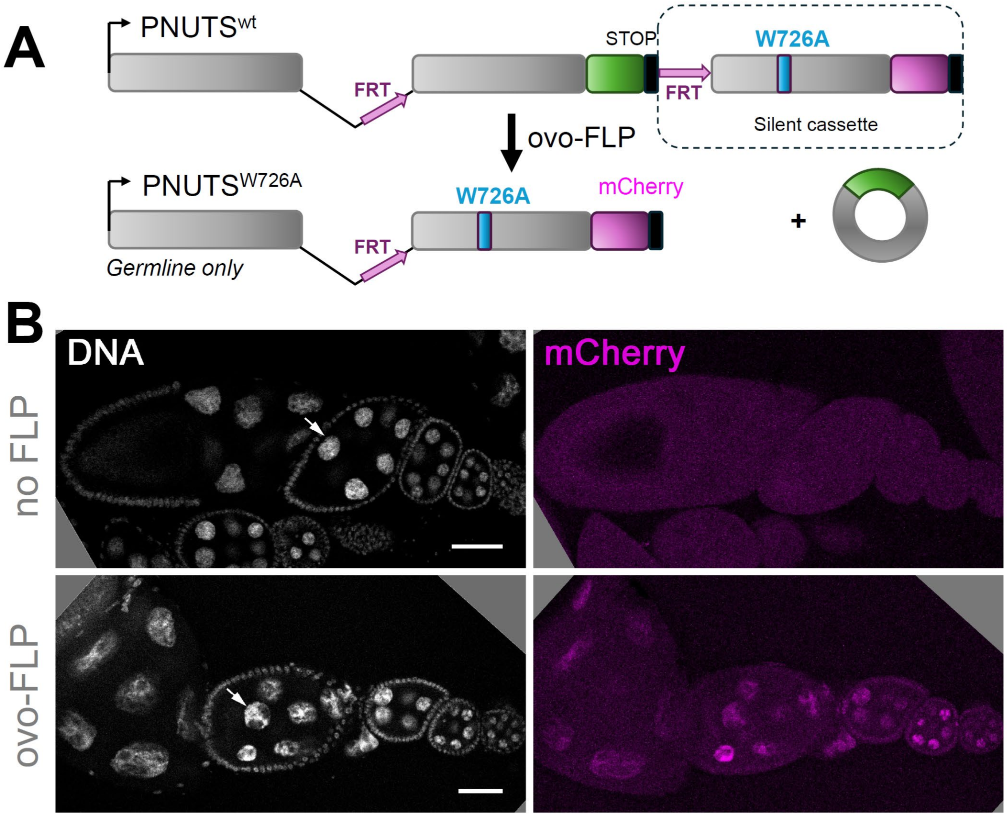

Figure S6. Conditional switching of PNUTS^{wt} to PNUTS^{W726A} in the *Drosophila* germline and associated phenotypes. A, Schematic of *PNUTS$^{wt\text{-}flp\text{-}W726A}$* construct design. Shown is an overview of 5' and 3' *PNUTS* coding exons separated by a large intron. 3' exon is flanked by Flp Recombination Target (FRT) sites that undergo recombination and removal of the wild type 3' exon in the presence of *ovo-FLP*, which is expressed specifically in the germline. The 3' exon is reconstituted by a downstream silent cassette carrying a W726A mutation and mCherry tag. **B,** confocal images showing egg chambers with or without *ovo-FLP*, stained for DNA. Nuclear mCherry is visible in germline nurse cells confirming allele exchange (wt to W726A). Arrows indicate nurse cell nuclei with PNUTS^{wt} (no FLP) and PNUTS^{W726A} (ovo-FLP) for comparison, showing non-dispersal of PNUTS^{W726A} chromosomes after stage 4. Scale bars, 50µm.

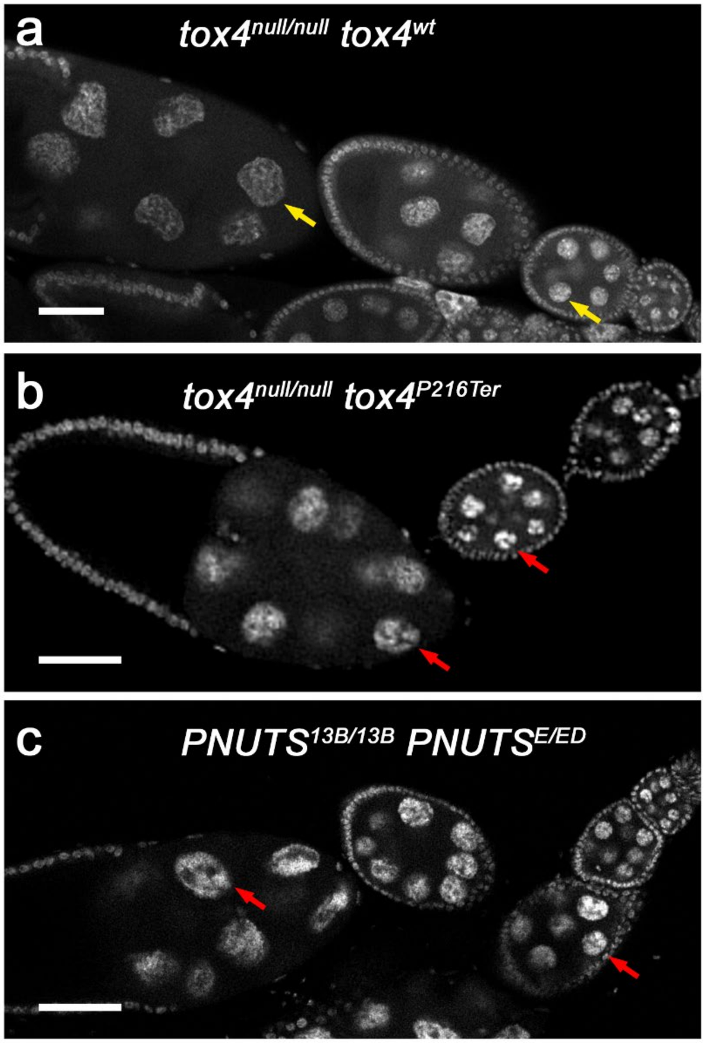

Figure S7. Chromosome dispersal phenotypes upon disruption of PNUTS-Tox4 binding. a-c, Shown are strings of egg chambers of different stages stained with DAPI, which labels both somatic follicular cell nuclei surrounding each chamber and large nurse cell germline nuclei. Genotypes are as indicated. **a**, Normal nurse cell chromosome dispersal (yellow arrows) is visible in a *tox4* null strain harbouring a wild type *tox4* genomic transgene. **b-c**, In contrast, dispersal defects, including condensed and ‘blobby’ chromosomes (exemplified by red arrows), are evident in (**b**) *tox4* or (**c**) *PNUTS* mutants that disrupt Tox4-PNUTS interactions (see main text for details). Scale bars, 50μm.
